# Defined Glycan Ligands for Detecting Rare l-Sugar-Binding Proteins

**DOI:** 10.1021/jacs.5c03251

**Published:** 2025-04-01

**Authors:** Hanee Kim, Tania J. Lupoli

**Affiliations:** Department of Chemistry, New York University, New York, New York 10003, United States

## Abstract

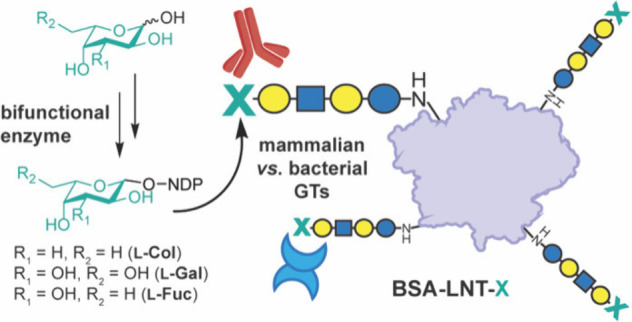

Most cells are decorated with distinct sugar sequences that can be recognized by carbohydrate-binding proteins (CBPs), such as antibodies and lectins. While humans utilize ten monosaccharide building blocks, bacteria biosynthesize hundreds of activated sugars to assemble diverse glycans. Monosaccharides absent in mammals are termed “rare” and are enriched in deoxy l-sugars beyond the “common” sugar l-fucose (l-Fuc) found across species. While immune proteins recognize microbial surfaces, there are limited probes to identify CBPs for the many rare sugars that may mediate these interactions. Here, we devise chemoenzymatic strategies to defined glycoconjugates containing l-Fuc and its structural analog l-colitose (l-Col), a bacterial dideoxysugar believed to bind immune proteins. We report a concise synthesis of l-Col and semisynthetic routes to several activated l-sugars. Incorporation of these sugars into glycans is evaluated using bacterial and mammalian glycosyltransferases (GTs) annotated to transfer l-Col or l-Fuc, respectively. We find that each GT can transfer both l-sugars, along with the rare hexose l-galactose (l-Gal), onto various glycan acceptors. Incorporation of these l-sugars into the resulting glycoconjugates is confirmed using known CBPs. Finally, these glycan ligands are employed to detect rare sugar-binding proteins in human serum. Overall, this work reveals similarities between bacterial and mammalian GTs that may be exploited for *in vitro* glycoconjugate construction to unveil novel mediators of host–pathogen interactions.

Cell surfaces contain glycans of varying sugar composition important for mediating cellular interactions.^1−5^ Within the bacterium *Escherichia coli* alone, ∼200 strains express distinct sugar sequences called O-antigens (O-Ags) found in lipopolysaccharides (LPSs) (Figure 1A).^6−8^ O-Ags can be distinguished by carbohydrate-binding proteins (CBPs), such as antibodies and lectins, similar to human blood group discrimination via surface glycan recognition.^9−12^ However, bacteria use hundreds of more sugar building blocks than mammals.^1,13,14^ Sugars absent in mammals are known as “prokaryote-specific” or “rare”^1,2^ and are enriched in deoxy l-sugars,^15^ including l-rhamnose (l-Rha), 6-deoxy-l-talose (l-6dTal), and l-colitose (l-Col), which is structurally related to the “common” sugar l-Fuc (Figure 1B).^8^l-Col is notably found on the termini of O-Ags in several pathogenic bacteria (e.g., *E. coli* strains O55 and O111),^8,16^ and immune CBPs are proposed to bind l-Col motifs.^17,18^ While select deoxy-l-sugar-recognizing antibodies are known,^19−22^ a lack of tools to study rare sugar recognition by proteins limits our understanding of their biological roles.^10^

**Figure 1:**
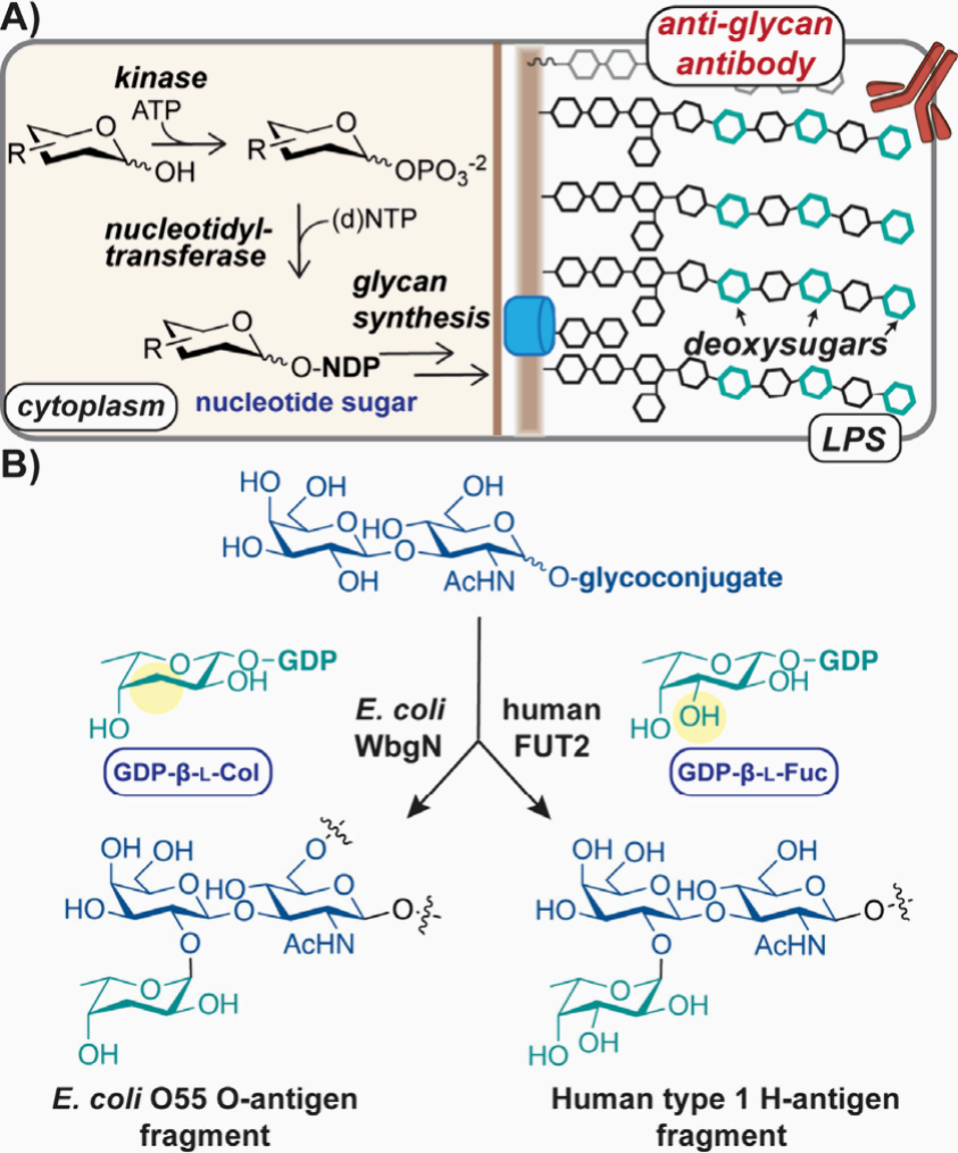
Cell surface glycans contain diverse deoxysugars. (A) Schematic of a bacterial cell envelope highlighting protein interactions with cell surface glycans in LPS. (B) Structurally related glycans from bacteria and humans contain l-Col and l-Fuc deoxy l-sugars, respectively.

Over the last two decades, lectin and antibody microarrays,^4,23^ sequencing-based approaches,^24^ and covalent capture-based methods^25^ have leveraged CBPs to help uncover the broad sequence diversity of cellular glycans.^10,26^ Conversely, glycan arrays^27−29^ have offered insight into the varying scopes of CBP recognition profiles.^30,31^ However, microbial glycan analytes are typically extracted from cells, which can complicate downstream analyses.^30,32,33^ While several bacterial surface glycans have been synthesized for vaccine development, schemes to uncommon sugar structures are often lengthy.^34,35^ It is also difficult to obtain rare nucleoside diphosphate (NDP)-sugar donors for chemoenzymatic strategies toward microbial glycoconjugates using glycosyltransferases (GTs);^36−47^ hence, there are gaps in our knowledge of bacterial glycan assembly,^8,48,49^ and new approaches are needed to probe the interactome of these glycans.^9,31,50−52^

Here, we aimed to create molecular tools for protein-based recognition of biologically relevant deoxysugars. We first optimized chemoenzymatic routes toward NDP-activated l-Col and l-Fuc, along with the structurally related rare hexose l-galactose (l-Gal). We then evaluated these molecules as substrates for GTs known to transfer activated l-Col (*E. coli* WbgN) or l-Fuc (human FUT2) in the assembly of two structurally related surface glycans in bacteria and humans, respectively (Figure 1B). Finally, we used these reagents to assemble defined glycoconjugates with different terminal l-sugars to detect interactions with known and novel CBPs.

While existing strategies could be optimized to activate commercially available l-Fuc,^53^ only a handful of schemes exist toward l-Col, which suffer from low overall yields.^54−58^ To avoid excessive protecting group manipulations, we devised a route to l-Col beginning with selective anomeric protection of l-Fuc with benzyl alcohol under acidic conditions^59,60^ leading to **1** (Schemes 1 and S1). To facilitate a Barton–McCombie deoxygenation, **1** was treated with *O*-phenyl chlorothionoformate in the presence of Oc_2_SnCl_2_, weak base, and additional catalyst^55^ to obtain the thiocarbonyl intermediate **2**, along with a small amount of double substituted side product (Figure S1). The organotin catalyst is known to coordinate *cis*-diols like the 3,4-hydroxyls of **1** for modification of C(3), as previously observed.^55,61^ In the next step, **2** was subjected to a radical-mediated deoxygenation^55^ to obtain **3**. Finally, various anomeric debenzylation conditions were tested, including hydrogenation of **3** with a Pd(II) catalyst,^60^ which reduced the anomeric aldehyde to form l-colitol (Figure S2).^62^ Consequently, **3** was instead deprotected using acidic cation exchange resin in water^58,63^ to yield a mixture of l-Col isomers (**4p**, **4f**) with the β-pyranose as the major product (Figure S3, Table S1).^58^ With an overall yield of 59% over four steps, this is currently the shortest reported synthesis of l-Col.

**Scheme 1:**
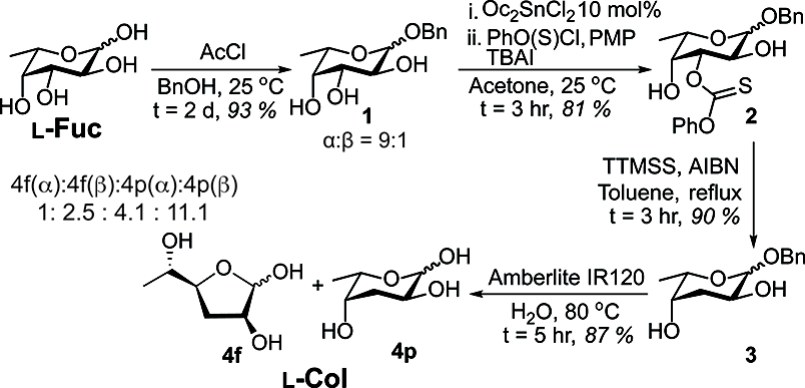
Chemical Synthesis of l-Col from l-Fuc Abbreviations: AcCl (acetyl chloride); BnOH (benzyl alcohol); Oc_2_SnCl_2_ (dioctyltin dichloride); PhO(S)Cl (*O*-phenyl chlorothionoformate); PMP (1,2,2,6,6-pentamethylpiperidine); TBAI (tetrabutylammonium iodide); TTMSS (tris(trimethylsilyl)silane); AlBN (azobisisobutyronitrile); **4p** (4-pyranose); **4f** (4-furanose).

l-Fuc and derivatives have been converted to their native GDP-β-l-sugar forms *in vitro* utilizing *Bacteroides fragilis*Fkp,^53,64^ which contains a C-terminal kinase domain (CTD) and an N-terminal guanylyltransferase domain (NTD) that utilize ATP and GTP, respectively (Figure 2A).^64−69^ HPLC/MS analysis of Fkp reactions containing l-Fuc, l-Col, and l-Gal indicated formation of corresponding GDP-sugars (Figures 2B,C and S4), as shown previously.^53,64^ After scale-up, we obtained milligram quantities of GDP-β-l-Fuc, -l-Col, and -l-Gal with yields of ≥50%.

**Figure 2:**
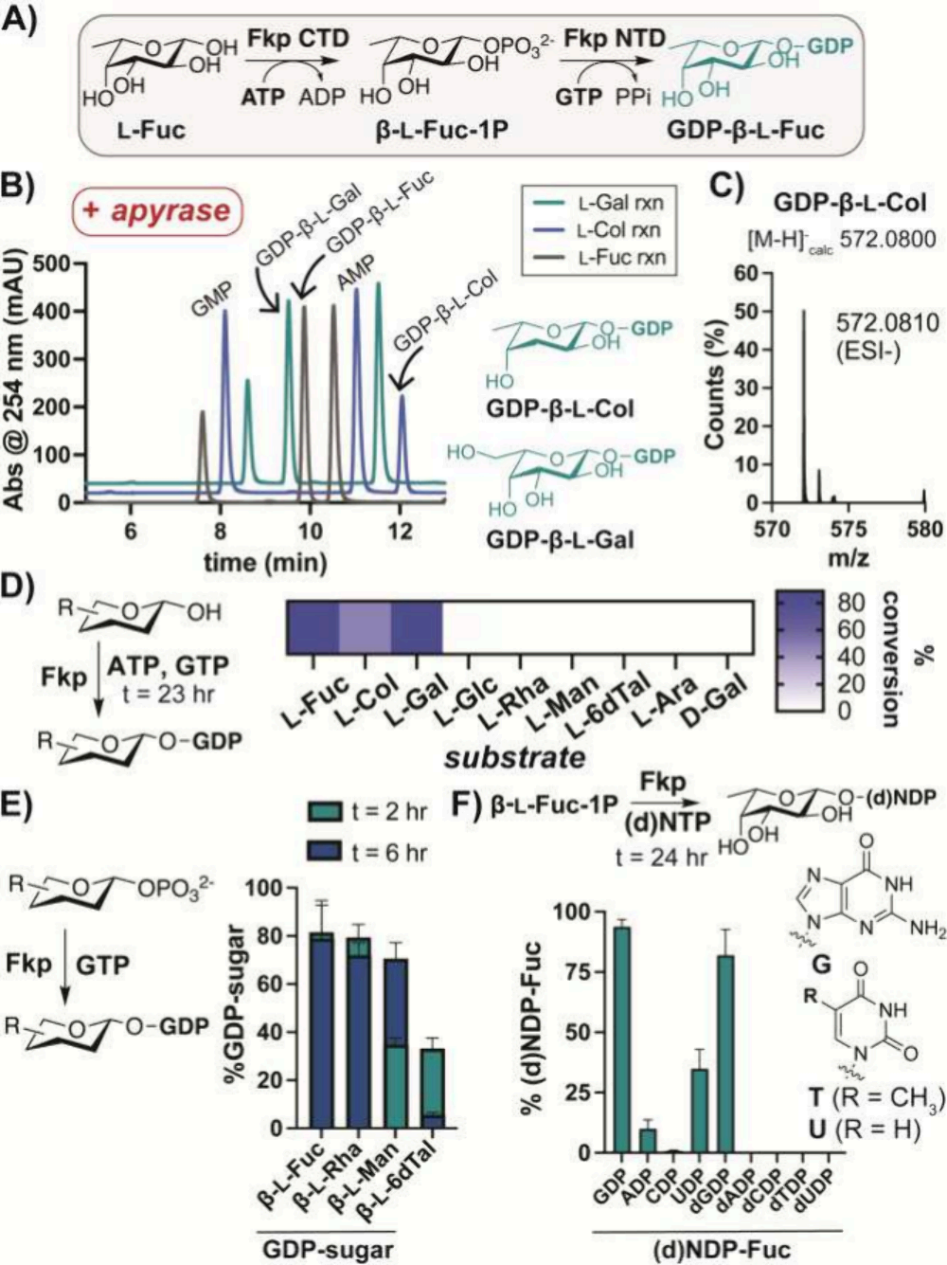
Fkp produces nucleotide-l-sugars. (A) Scheme of bifunctional enzyme Fkp. (B) HPLC analysis of activated l-Fuc, l-Col, and l-Gal by Fkp following apyrase-mediated hydrolysis of NDPs/NTPs. (C) Representative HRMS analysis confirms GDP-β-l-Col production. (D) Monosaccharide scan indicates l-Fuc analogs are preferred by Fkp. (E) Activation of β-l-sugar-1Ps demonstrates promiscuity in nucleotidyl transfer. (F) Nucleotide scan demonstrates a preference of Fkp for (d)GTP. Bars indicate standard deviation (SD), *n* = 3–9.

While Fkp offers a convenient route to analogs of GDP-β-l-Fuc, we observed that other monosaccharides were not activated (Figures 2D and S4, Table S2). Notably, inversion of 2-OH (l-6dTal) or 4-OH (l-Glc) prevented turnover. We reasoned that the kinase domain might limit the substrate scope, as nucleotidyltransferases often exhibit promiscuity.^43,44,70−73^ Accordingly, we observed Fkp could directly activate β-l-Fuc-1-phosphate (β-l-Fuc-1P), as well as β-l-Rha/6dTal/Man-1P synthesized by published routes^44^ (Figures 2E and S5, Table S3). Notably, hydrolysis of GDP-β-l-6dTal was reduced by using shorter reaction times (Figure S5B). Further, evaluation of various nucleotides as substrates demonstrated ≥10% yields with only GTP, ATP, UTP, and dGTP (Figures 2F and S6, Table S4). UTP has H-bond capabilities similar to GTP that may promote binding, highlighting substrate flexibility not previously observed for Fkp.

Using this collection of activated analogs, l-sugar incorporation into biomolecules by representative GTs could be evaluated. We focused on *E. coli* WbgN and human FUT2 of the GT-11 family^74^ because both utilize GDP-β-l-deoxysugar donors and similar glycan acceptors (Figure 1B).^53,75,76^ Further, alignment of WbgN and FUT2 models^77^ indicated high structural similarity (Figure S7), suggesting shared biochemical characteristics. To assess their substrate scopes, HPLC/MS analyses of each GT was performed with various donors and a lacto-*N*-biose (LNB) acceptor. We observed that both utilize GDP-β-l-Col or (d)GDP-β-l-Fuc, indicating that variation at the sugar C(3) or the ribose C(2′) was tolerated (Figures 3A, S8, and S9, Table S5). GDP-β-l-Gal was also consumed, demonstrating that a 6-OH is not excluded; however, GDP-sugars with altered hydroxyl stereochemistry (e.g., l-Rha/Man) or nucleotides (e.g., UDP, ADP) were not utilized as donors by either GT. Despite these similarities, the WbgN reaction was metal-independent,^53^ while FUT2 showed metal-dependency (Figure S10).

**Figure 3:**
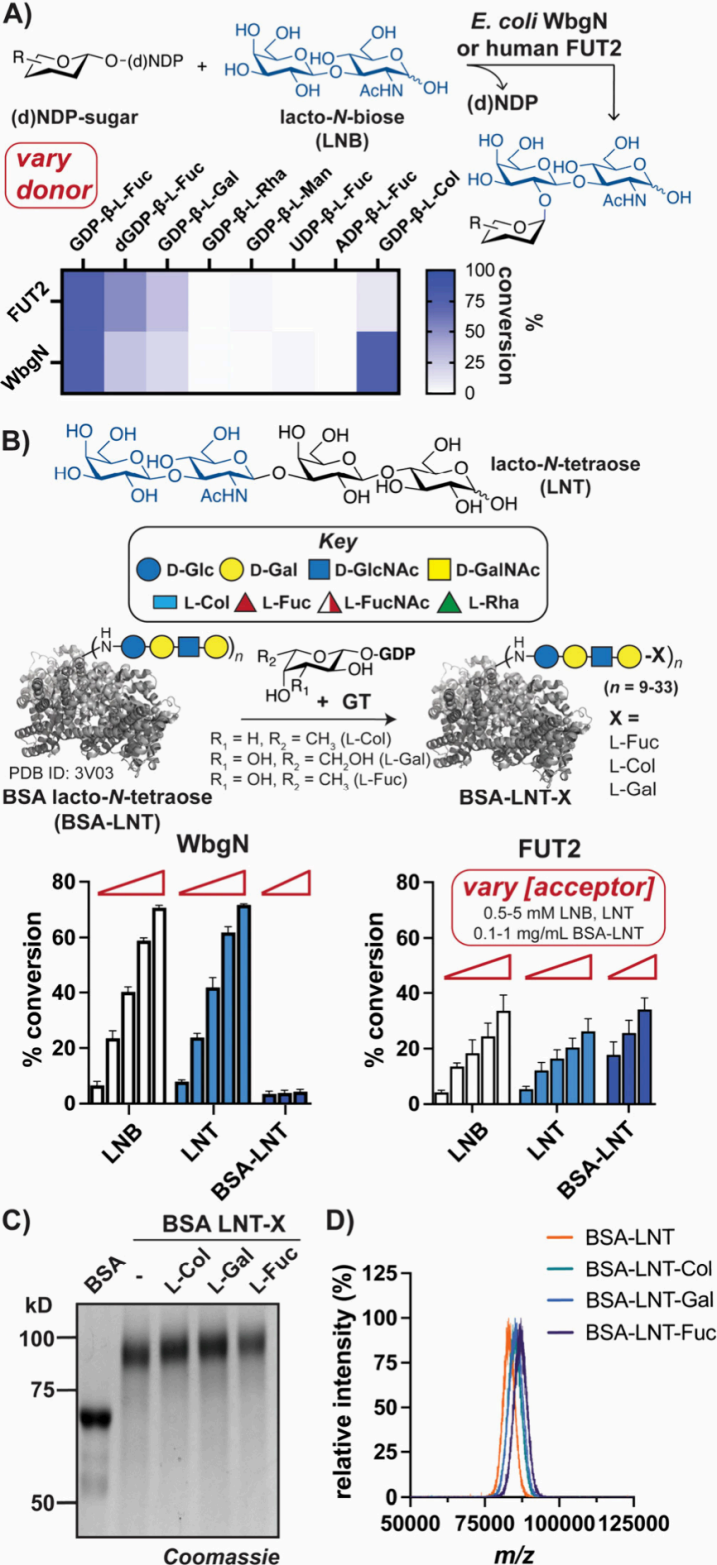
Bacterial and human GTs transfer l-sugars onto various glycans. (A) Donor analysis of GTs indicates transfer of l-Col, l-Fuc, and l-Gal onto LNB. (B) Schematic of l-sugar glycoconjugate semisynthesis (top). Analysis of GTs with native donors and increasing [acceptors] indicates FUT2 labels glycoproteins more efficiently (bottom). Increased MW upon l-sugar addition onto BSA-LNT by FUT2 observed by (C) SDS-PAGE and (D) MALDI-TOF MS analyses (Figure S12). Bars indicate SD, *n* = 3–9.

To generate glycan-based probes for CBP detection, we next evaluated alternate acceptor substrates.^78−80^ We postulated that an available glycoprotein, BSA lacto-*N*-tetraose (BSA-LNT), could serve as an acceptor, as the LNT contains a terminal LNB disaccharide. We first compared native donor transfer by each GT onto varying concentrations of LNB, LNT, and BSA-LNT (Figure 3B, Table S6). While WbgN modified free LNT and LNB in high yields, FUT2 more efficiently labeled glycoprotein. Notably, FUT2 uses cellular glycoprotein acceptors,^75,76,81^ while WbgN modifies glycolipids.^8,53^ To enable comparison of structurally related l-sugar scaffolds, FUT2-mediated transfer of l-Fuc, l-Col, and l-Gal onto BSA-LNT was evaluated. SDS-PAGE analysis indicated a MW increase in each reaction (Figure 3C); further, HPLC and MALDI MS analyses revealed ∼12–24 added l-sugars per BSA-LNT, which contain 9–33 LNT each (Figures 3D, S11, and S12).

To characterize our glycoprotein probes, blood group antibodies were initially used, as LNT mimics blood antigen precursors, and fucosylated LNB is the terminal sequence motif of type 1 H-antigens, a major soluble human blood group antigen (Figure 1B).^12,82^ Immunoblot analysis with an anti-LNT antibody confirmed detection of BSA-LNT, but not l-sugar-labeled glycoproteins (Figures 4A and S13A,B), as further supported by ELISA analysis (Figure S13C). Accordingly, anti-H-antigen (type 1) antibody detected l-Fuc glycoconjugates with higher sensitivity than those with terminal l-Col or l-Gal (Figures 4B and S14). As a comparison, we assessed recognition using an antibody for type 2 H-antigen,^12^ which differs from type 1 by a single glycosidic linkage (Figure 4C, top); however, none of the glycoconjugates were detected (Figure S15), presumably due to their backbone structures.

**Figure 4:**
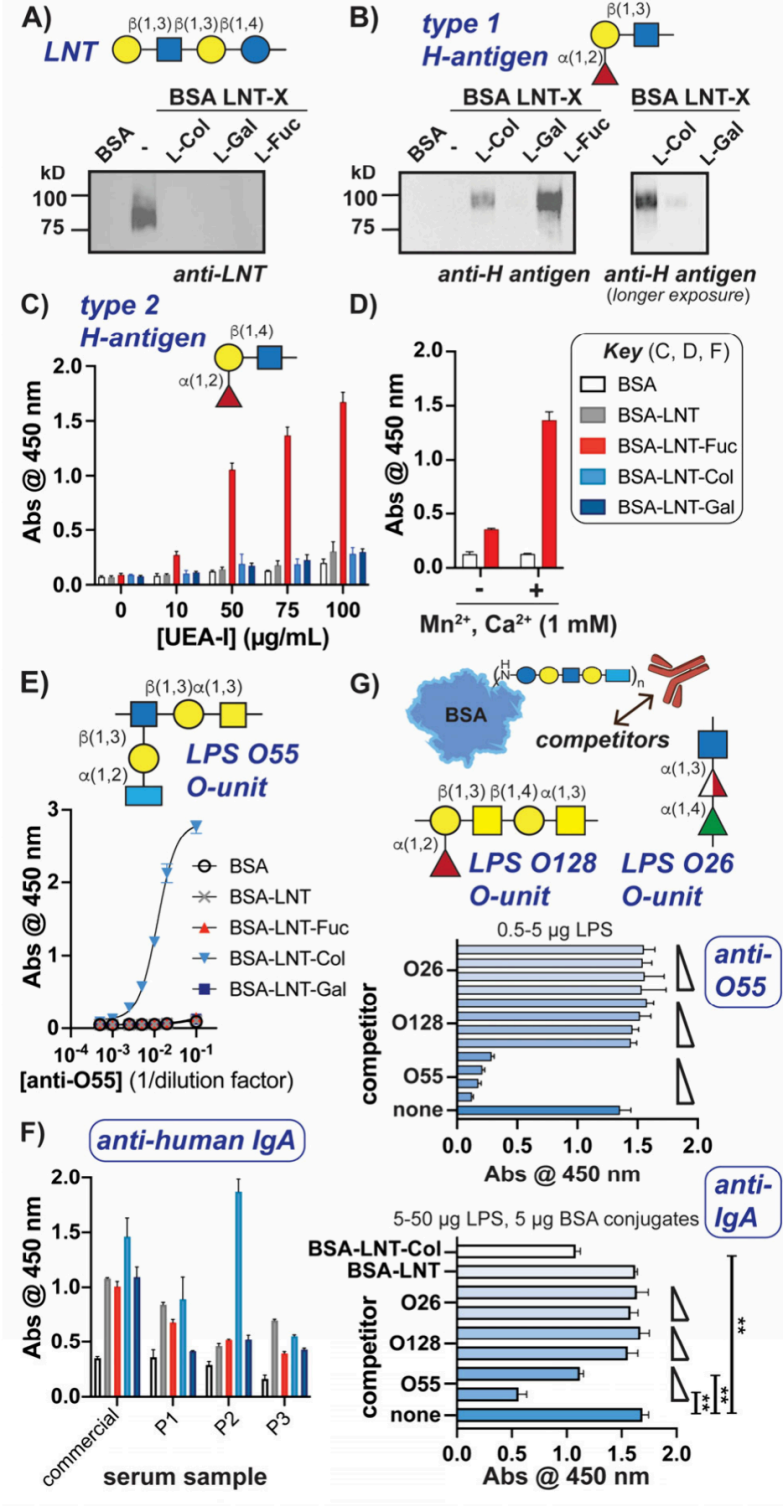
Defined glycoconjugates detect l-sugar-binding CBPs. (A, B) Immunoblot analyses with indicated primary antibodies confirm l-sugar-labeling of BSA-LNT. (C, D) Lectin ELISA analysis detects terminal l-Fuc in a metal-dependent manner. (E) Anti-LPS O55 reacts only with l-Col glycan. (F) ELISA analysis of commercial and clinical (P1–P3) human serum samples indicates varying levels of anti-l-Col IgA antibodies. (G) Competition assays show specificity of BSA-LNT-Col interactions with anti-O55 and IgA from P2 (alternate O128 structures shown in Figure S17). **p < 0.0021, paired *t* test used; bars indicate SD for *n* = 3.

Lectins are also immune CBPs known to recognize eukaryotic and microbial glycans.^9,11,83^ A well-characterized plant lectin, *Ulex europaeus* agglutinin-I (UEA-I), has been shown to primarily recognize l-Fuc on type 2 H-antigens in a metal-dependent fashion.^52,84,85^ Interestingly, direct ELISA analysis indicated that UEA-I could still detect l-Fuc in the type 1 glycan mimic, but not other terminal l-sugars, and that binding was metal-dependent (Figure 4C,D).

To further benchmark our probes, we next enlisted antibodies used for microbial strain serotyping. The LNB-l-Col sequence mimics a bacterial O55 oligosaccharide unit (O-unit) fragment, which lacks l-Fuc and l-Gal (Figure 1B); accordingly, anti-O55 antibody reacted only with BSA-LNT-l-Col, but not the other glycoproteins (Figure 4E). Overall, these data indicate that l-sugars were indeed added to the terminal position of BSA-LNT and are accessible to known CBPs.

Finally, we sought to assess whether the probes could detect rare sugar CBPs in biologically relevant samples. We analyzed commercial and clinical human serum samples with our panel of glycoconjugate probes and compared levels of IgM, IgG, and IgA antibodies (Figures 4F and S16A,B), as these are the most common isotypes in human serum.^31,86^ We observed that l-Col binding was significantly enriched in IgA fractions, especially in patient serum sample 2 (P2). In line with this observation, serum and mucosal IgA are known to bind microbes as part of protective mechanisms.^86−89^

Specificity for l-Col binding was validated by competition experiments using O55 LPS and two LPSs containing other deoxy l-sugars, O26 (l-Rha and l-FucNAc) and O128 (l-Fuc). We first demonstrated that anti-O55 antibody was competed from BSA-LNT-l-Col analyte in a concentration-dependent manner by O55 LPS, but not by O26 or O128 (Figure 4G, top). Similarly, the interaction between clinical IgA antibodies (P2) and BSA-LNT-l-Col could only be disrupted by O55 LPS or BSA-LNT-l-Col, not by other LPSs, BSA-LNT (Figure 4G, bottom), or excess free sugars (Figure S16C). Hence, l-Col-recognizing antibodies exist in human serum.

In conclusion, we developed concise routes to nucleotide l-sugars that offer a useful alternative to enzyme cascade approaches^90,91^ and provide substrates for GTs to produce various glycans. This strategy avoids known challenges with the chemical glycosylation of deoxysugars.^20^ Similar to our findings, other fucosyltransferases can utilize C(6)-modified sugar donors,^92^ including activated l-Gal in plants.^93^ The ability of FUT2 and WbgN to transfer rare and common sugars to different acceptors emphasizes the importance of substrate availability in dictating natural glycan sequences. Indeed, our results reflect the inherent promiscuity of GTs,^94^ which facilitates metabolic oligosaccharide engineering,^2,95^ and may promote bacterial “mimicry” of host glycans.^96^ In fact, nonhuman sugars have been observed in human glycans,^97,98^ and host-like microbial glycans can stimulate an immune response.^10^ As demonstrated here, this promiscuity of GTs can also be leveraged *in vitro* to construct defined glycoconjugates that vary only in the degree of hydroxylation of a *single* sugar. Hence, important sugar binding motifs for detected CBPs can be rapidly determined. Notably, these glycoproteins lack the sequence space and valencies of native architectures.^25^ Nonetheless, similar to our observations, synthetic trisaccharide rare sugar antigens recently uncovered anti-l-Col IgA antibodies across human breast milk samples.^20^ We envision that evolution approaches may be employed to expand the scope of enzymes towards new glycoconjugate probes. Consequently, in addition to receptors for l-Col and l-Gal, we expect other molecular players will soon be unveiled that mediate the interplay between bacteria and their hosts.

## Supporting Information Available

Tables S1–S6, Scheme S1, and Figures S1–S17; description of experimental methods for syntheses; compound characterization (NMR and MS analyses); methods for molecular cloning, protein expression, and purification, along with biochemical assays using Fkp and GTs (HPLC and MS analyses); Tables S7–S10 containing information on plasmids, primers, strains, and CBPs that were utilized (PDF)

## Abbreviations

Carbohydrate-binding protein: CBP
l-fucose: l-Fuc
l-colitose: l-Col
l-rhamnose: l-Rha
6-deoxy-l-talose: l-6dTal
l-galactose: l-Gal
glucose: Glc
*N*-acetyl glucosamine: GlcNAc
mannose: Man
*N-*acetyl-l-fucosamine: l-FucNAc
glycosyltransferase: GT
O-antigen: O-Ag
high-resolution mass spectrometry: HRMS
time-of-flight: TOF
high-pressure liquid chromatography: HPLC
bovine serum albumin lacto-*N*-tetraose: BSA-LNT
matrix-assisted laser desorption/ionization: MALDI
enzyme-linked immunosorbent assay: ELISA
