# Supplementary material for: Defined Glycan Ligands for Detecting Rare l-Sugar-Binding Proteins
Source: J Am Chem Soc. 2025 Apr 1;147(14):11693–9. doi: 10.1021/jacs.5c03251 (PMC11987014; doi:10.1021/jacs.5c03251)
Supplement: Supplementary file 1 — ja5c03251_si_001.pdf [file ja5c03251_si_001.pdf]

Supplemental Information

Defined Glycan Ligands for Detecting Rare L-Sugar-Binding Proteins

Hanee Kim, Tania J. Lupoli*

Department of Chemistry, New York University, New York, NY, 10003

*Email: tjl229@nyu.edu

Experimental Synthetic Procedures and Data

General information for synthetic compounds. All solvents were purchased from Fisher Scientific or Sigma Aldrich. All chemicals were purchased from Fisher Scientific, Sigma Aldrich, Alfa Aesar, TCI chemicals or Chem-Impex International (L-fucose) and used without further purification. All reactions were performed under nitrogen atmosphere. Flash column chromatography was performed on silica gel 60 (230-400 mesh) from Fisher Scientific. Analytical thin layer chromatography was performed on silica gel 60 F_{254} aluminum plates, which were visualized under UV and/or by staining with ceric ammonium molybdate (CAM) followed by brief heating. NMR spectra were obtained using a Bruker Avance III HD 400 NMR Spectrometer at 400 MHz for ^{1}H NMR and 101 MHz for ^{13}C NMR. All NMR spectra were reported in parts per million (ppm), and calibrated using residual non-deuterated solvent as an internal reference, followed by processing by MestReNova (authorized to NYU). Homonuclear correlation spectroscopy (COSY) and heteronuclear single-quantum correlation spectroscopy (HSQC) were used for proton assignments. Nuclear Overhauser effect spectroscopy (NOESY) was used for anomeric configuration determination. Distortionless enhancement by polarization transfer (DEPT) techniques were also used to analyze each compound. High-resolution mass spectrometry (HRMS) analyses were acquired on an Agilent 6224 Accurate-Mass Time-of-Flight LC/MS (LC-TOF) spectrometer with an electrospray (ESI) ionization source equipped with an autosampler.

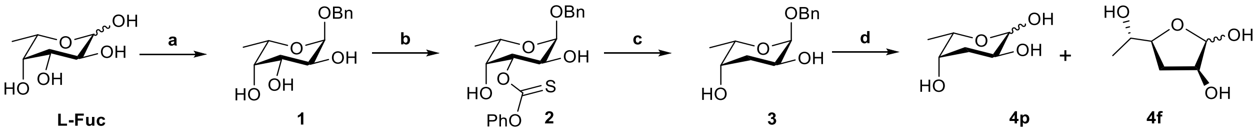

Scheme S1. Synthesis of L-Colitose (4). a) AcCl, BnOH, 2 d, 25 °C; b) (1) 10 % Oc_2SnCl_2, 25 °C, (2) PhOSCl, PMP, TBAI, acetone, 3 h, 25 °C; c) TTMSS, AIBN, toluene, reflux, 3 h; d) Amberlite IR-120, water, 5 h, 80 °C.

Synthesis of Benzyl-L-fucopyranoside (**1**)[1-2]

L-Fucose (1.5 g, 9.1 mmol) was suspended in benzyl alcohol (BnOH, 18 mL), while acetyl chloride (AcCl, 0.35 mL, 4.9 mmol) was added to the mixture dropwise. The reaction was allowed to proceed for t = 2 d at 25 °C with constant stirring. The reaction was diluted with 40 mL chloroform and neutralized with 10 g of solid $NaHCO_3$. The reaction was filtered and concentrated under reduced pressure at 50 °C. The crude reaction was subjected to column chromatography (100 % ethyl acetate (EtOAc) to 10 % MeOH in EtOAc) to afford 2.2 g of **1** (α:β=9:1) as a white solid with a yield of 93 %, which was directly used in the next step without further purification. Notably, when the reaction was carried out at 40 °C, β-product was favored, but with decreased yield. The reaction did not proceed at 0 °C.

α-anomer: ^{1}H NMR (400 MHz, CD_3CN) δ 7.42 – 7.31 (m, 5H, Ph*H*), 4.83 (d, J = 3.7 Hz, 1H, *H-1*), 4.69 (d, J = 11.9 Hz, 1H, -OC*H*$_2$Ph), 4.49 (d, J = 11.9 Hz, 1H, -OC*H*$_2$Ph), 3.97 – 3.89 (m, 1H, *H-5*), 3.69 – 3.54 (m, 3H, *H-2, H-3*, and *H-4*), 3.07 (d, J = 5.6 Hz, 1H, O*H*), 2.88 (d, J = 4.0 Hz, 1H, O*H*), 2.69 (d, J = 8.3 Hz, 1H, O*H*), 1.15 (d, J = 6.6 Hz, 3H, *H-6*) ppm; ^{13}C NMR (101 MHz, CD_3CN) δ 139.2, 129.2, 128.8, 128.5, 99.4, 72.8, 71.5, 70.0, 67.1, 16.6 ppm; DEPT-135 (101 MHz, CD_3CN) δ 129.1, 128.8, 128.4, 99.3, 72.7, 71.4, 69.9, 69.8, 67.0, 16.5 ppm; HRMS m/z: $[M+Na]^+$ Calcd. for $C_{13}H_{18}O_5Na$ 277.1046; found: 277.1041; deviation: 1.99 ppm.

Synthesis of Benzyl 3-*O*-phenoxythiocarbonyl-α-L-fucopyranoside (**2**)[3-4]

To prepare anhydrous acetone, heated molecular sieves (4 Å beads, 4-8 mesh) were slowly added to a round bottom flask containing acetone under N_2 prior to use (t = 30 min prior to reaction). In a foil-covered round bottom flask, compound **1** (1.6 g, 6.3 mmol) and dioctyltin dichloride (Oc_2SnCl_2, 0.26 g, 0.62 mmol) were suspended in anhydrous acetone (60 mL) and stirred for t = 15 min at 25 °C. Then, tetrabutylammonium iodide (TBAI, 0.70 g, 1.9 mmol), *O*-phenyl chlorothionoformate (PhO(S)Cl, 1.4 mL, 10 mmol), and 1,2,2,6,6-pentamethylpiperidine (PMP, 1.8 mL, 9.9 mmol) were added to the reaction mixture, and the reaction was stirred for t = 3 h at 25 °C. The reaction was quenched with an equal volume of saturated NH_4Cl and extracted three times with EtOAc. The combined organic layer was then washed with water and brine, dried over Na_2SO_4, filtered, and concentrated under reduced pressure at <20 °C. The crude reaction was subjected to column chromatography (20% EtOAc in hexane to 40% EtOAc in hexane) to afford 2.1 g of **2** (α-anomer) as a pale-yellow oil in 81 % yield. The double-substituted **2s** (α:β=3:1, **Figure S1**) was also obtained with a 5.4 % yield as a side product.

^{1}H NMR (400 MHz, $CDCl_3$) δ 7.47 – 7.27 (m, 8H, Ph*H*), 7.14 (dt, J = 7.6, 1.2 Hz, 2H, Ph*H*), 5.62 (dd, J = 10.3, 3.0 Hz, 1H, *H-3*), 5.08 (d, J = 4.0 Hz, 1H, *H-1*), 4.79 (d, J = 11.5 Hz, 1H, -OCH_2Ph), 4.62 (d, J = 11.9 Hz, 1H, -OCH_2Ph), 4.23 (dd, J = 10.4, 4.1 Hz, 1H, *H-2*), 4.17 (d, J = 3.0 Hz, 1H, *H-4*), 4.10 (q, J = 6.7 Hz, 1H, *H-5*), 1.29 (d, J = 6.6 Hz, 3H, *H-6*) ppm; ^{13}C NMR (101 MHz, $CDCl_3$) δ 195.2, 153.5, 137.0, 129.7, 128.8, 128.3, 128.3, 126.9, 122.0, 98.2, 84.1, 70.3, 70.0, 67.2, 66.2, 16.1 ppm; DEPT-135 (101 MHz, $CDCl_3$) δ 129.5, 128.6, 128.1, 126.7, 121.8, 98.0, 83.9, 70.1, 69.8, 67.0, 66.0, 15.9 ppm; HRMS m/z: $[M+Na]^+$ Calcd. for $C_{20}H_{22}O_6SNa$ 413.1029; found: 413.1022; deviation: 1.76 ppm.

Synthesis of Benzyl 3,6-dideoxy-α-L-*xylo*-hexopyranoside (**3**)[3]

In a foil-covered round bottom flask, compound **2** (0.24 g, 0.61 mmol), tris(trimethylsilyl)silane (TTMSS, 0.37 mL, 1.2 mmol) and azobisisobutyronitrile (AIBN, 0.020 g, 0.12 mmol) were refluxed at 110 °C in anhydrous toluene (24 mL) with constant stirring for t = 3 h. The use of TTMSS in place of tributyltin hydride improved the yield by 2-fold, similar to previous reports.[3] The reaction was concentrated under reduced pressure, and the crude sample was subjected to column chromatography (50% EtOAc in hexane (Hex) to 70% EtOAc in Hex) to afford 0.13 g of **3** as a pale-yellow oil in 90 % yield.

^{1}H NMR (400 MHz, MeOD) δ 7.46 – 7.25 (m, 5H, Ph*H*), 4.81 (d, J = 3.6 Hz, 1H, *H-1*), 4.74 (d, J = 12.1 Hz, 1H, -OCH_2Ph), 4.60 (d, J = 12.0 Hz, 1H, -OCH_2Ph), 4.06 – 3.96 (m, 1H, *H-2*), 3.91 (qd, J = 6.6, 1.5 Hz, 1H, *H-5*), 3.71 (dd, J = 4.2, 2.2 Hz, 1H, *H-4*), 2.08 – 1.97 (m, 1H, *H-3*), 1.97 – 1.88 (m, 1H, *H-3*), 1.15 (d, J = 6.6 Hz, 3H, *H-6*) ppm; ^{13}C NMR (101 MHz, MeOD) δ 139.4, 129.3, 129.1, 128.6, 99.3, 70.4, 69.9, 67.4, 64.8, 35.6, 16.6 ppm; DEPT-135 (101 MHz, MeOD) δ 129.0, 128.8, 128.3, 99.0, 70.1, 69.7, 67.2, 64.5, 35.3, 16.3 ppm; HRMS m/z: $[M+NH_4]^+$ Calcd. for $C_{13}H_{18}O_4NH_4$ 256.1543; found: 256.1538; deviation: 2.03 ppm.

Synthesis of L-Colitose (**4**)[5-6]

A solution of compound **3** (0.39 g, 1.6 mmol) in water (4 mL) was treated with ion exchange IR-120 hydrogen form resin (0.78 g) and stirred at 80 °C for t = 5 h. The reaction was then filtered and concentrated under reduced pressure. The crude reaction was subjected to column chromatography on triethylamine (TEA)-deactivated silica (2.5 % TEA in 1:4 Hex:EtOAc) to afford 0.21 g of an isomeric mixture of **4** (L-colitose · TEA) as a yellow oil in 87 % yield.

By comparison to the ^{1}H-NMR spectrum that was previously reported,[5] the equilibrium ratio in water was as follows (**Table S1**):

α-pyranose (**4p**-α): β-pyranose (**4p**-β): α-furanose (**4f**-α): β-furanose (**4f**-β)=4.1: 11.1: 1: 2.5

β-L-Colitopyranose (**4p**-β, major isomer):

^{1}H NMR (400 MHz, D_2O) δ 4.54 (d, J = 8.0 Hz, 1H, *H-1*), 3.87 – 3.78 (m, 2H, *H-4*, *H-5*), 3.63 (ddt, J = 12.5, 8.0, 4.1 Hz, 1H, *H-2*), 3.20 (CH_2 of TEA), 2.23 – 2.17 (m, 1H, *H-3*), 1.76 – 1.68 (m, 1H, *H-3*), 1.28 (CH_3 of TEA), 1.20 (d, J = 6.5 Hz, 4H, *H-6* of mixture) ppm; ^{13}C NMR (101 MHz, D_2O) δ 98.7, 74.7, 68.9, 66.9, 37.6, 16.3 ppm; DEPT-135 (101 MHz, D_2O) δ 92.1, 74.7, 68.9, 66.9, 47.2 (*C*H_2 of TEA), 37.6, 16.3, 8.8 (*C*H_3 of TEA) ppm; HRMS m/z: $[M+Na]^+$ Calcd. for $C_6H_{12}O_4Na$ 171.0628; found: 171.0623; deviation: 2.58 ppm. HSQC reported in **Figure S3**.

Minor isomers (**4p**-α, **4f**-α, and **4f**-β; only anomeric positions indicated):

^{1}H NMR (400 MHz, D_2O) δ 5.27 (d, J = 1.2 Hz, 0.2H, *H-1* of β-furanose), 5.20 (d, J = 4.4 Hz, 0.1H, *H-1* of α-furanose), 5.13 (d, J = 3.7 Hz, 0.4H, *H-1* of α-pyranose) ppm; ^{13}C NMR (101 MHz, D_2O) δ 102.7 (C-1 of β-furanose), 95.7 (C-1 of α-furanose), 92.1 (C-1 of α-pyranose) ppm.

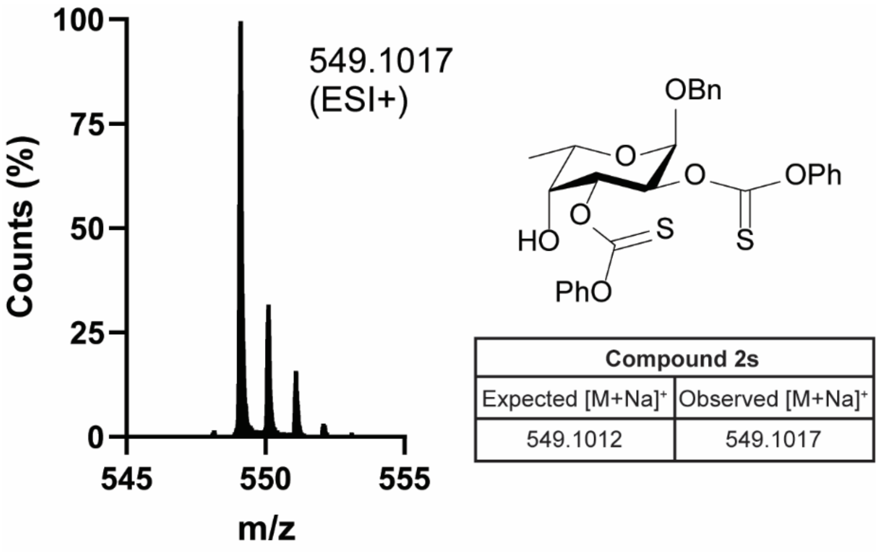

Compound 2s	
Expected [M+Na]+	Observed [M+Na]+
549.1012	549.1017

Figure S1. Characterization of 2s as a side-product by HRMS. During the reaction to synthesize compound **2**, formation of the C-2 and C-3 double-substituted side product (compound **2s**) was confirmed by HRMS with 5.4 % yield.

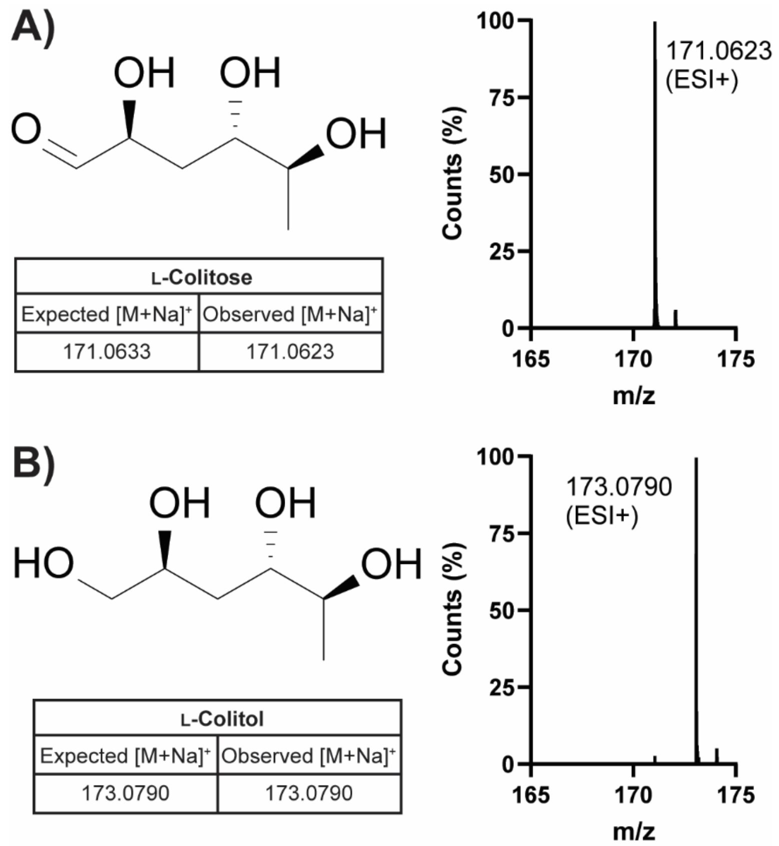

Figure S2. Characterization of L-colitol produced under strong reducing conditions by high-resolution mass spectrometry (HRMS). A) Anomeric debenzylation of **3** using acidic cation exchange resin in water resulted in formation of L-colitose (**4**) as indicated by HRMS. B) The use of Pd (II) catalyst resulted in reduction of the aldehyde to an alcohol, forming L-colitol, which was indicated by HRMS.

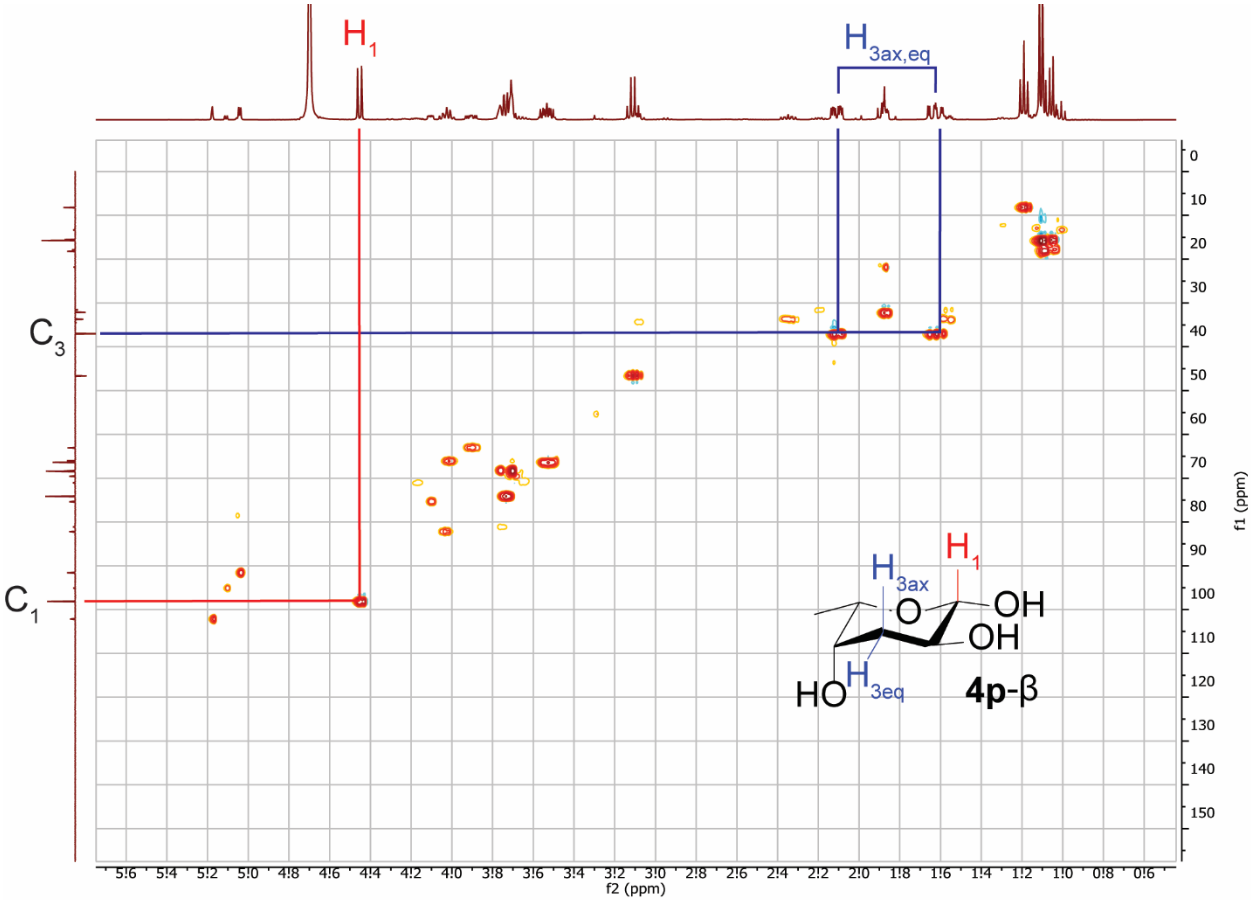

Figure S3. HSQC spectrum of β-L-colitopyranose (4p-β, major isomer). C-3 (DEPT-135 spectrum) shows correlation with two H-3 protons in the ^1H NMR spectrum (blue). Anomeric C-1 shows correlation with H-1 of β-pyranose (red).

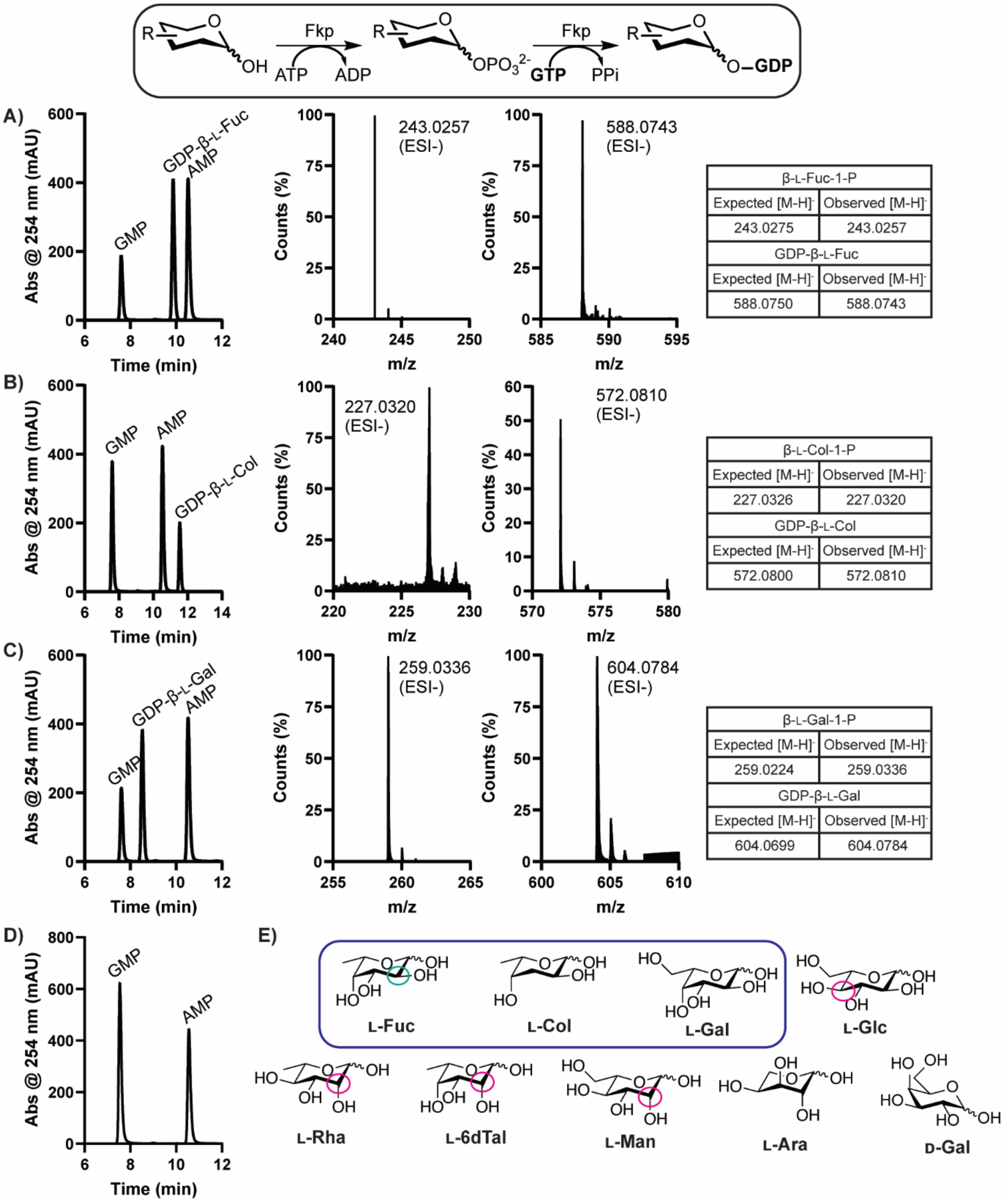

Figure S4. Characterization of each GDP-β-L-sugar produced by Fkp using HPLC and HRMS. HPLC and HRMS analyses of Fkp reactions with A) L-Fuc, B) L-Col, or C) L-Gal (10 mM each) in addition to 10 mM ATP and GTP. The production of each β-L-S-1P (middle) and GDP-β-L-sugar (right) was confirmed by HRMS. HPLC analysis of GDP-β-L-sugars was performed after treating each reaction with apyrase, which converted the remaining nucleotides to AMP and GMP (left). D) HPLC trace of GMP and AMP standards. E) Chemical structures of L/D-sugars used in **Figure 2D**. Fkp activates structural analogs of L-Fuc (box) with C(2) indicated with a green circle. Red circles indicate representative hydroxyl stereochemistry that differs from the native substrate L-Fuc.

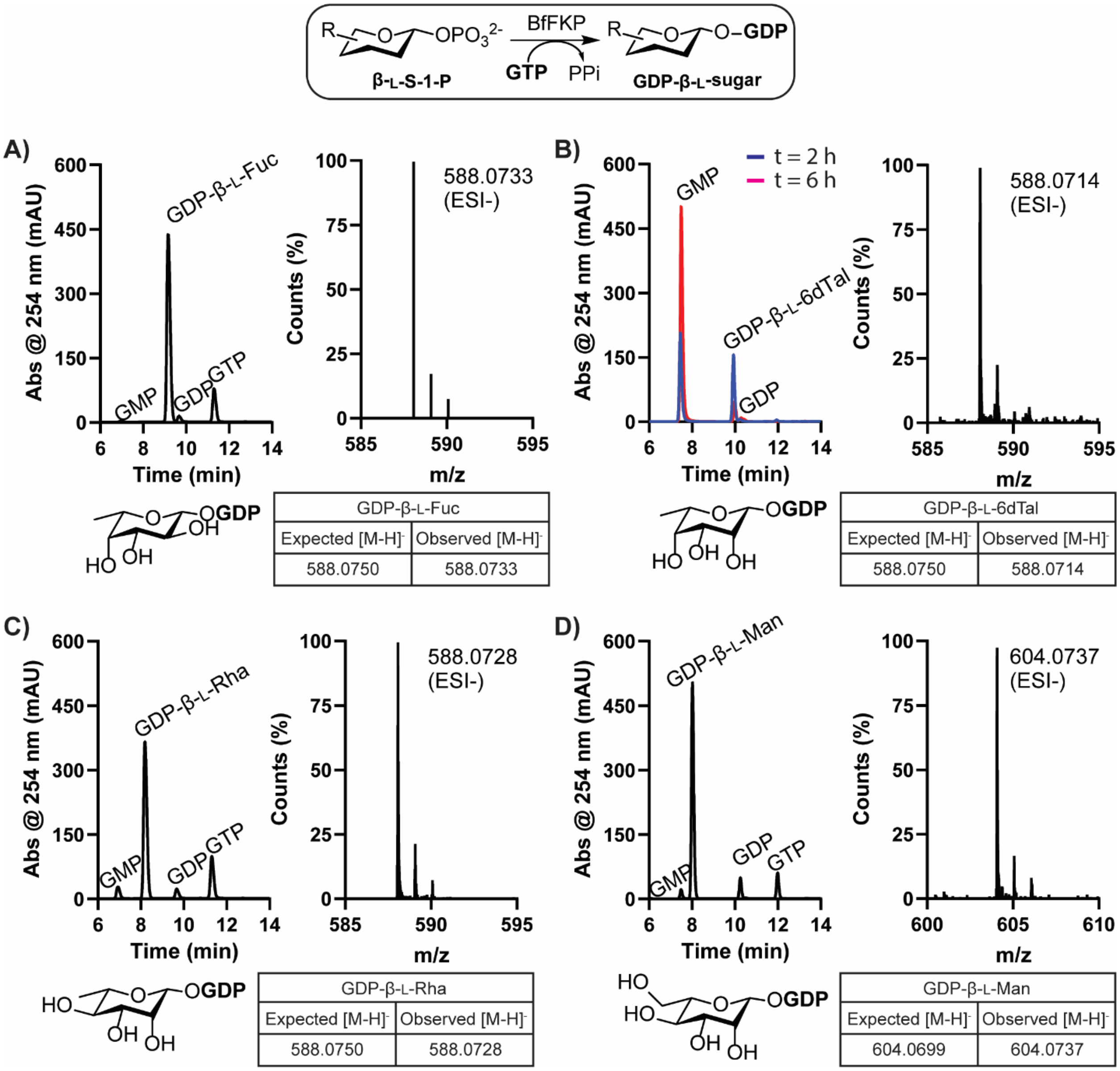

Figure S5. Characterization of each GDP-β-L-sugar produced by Fkp with different β-L-sugar-1Ps using HPLC and HRMS. Reactions containing A) β-L-Fuc-1P, B) β-L-6dTal-1P, C) β-L-Rha-1P, and D) β-L-Man-1P (10 mM each) with 10 mM GTP each were converted to corresponding GDP-sugars by Fkp as indicated by HPLC (left) and HRMS analysis (right). In part B, a longer reaction time for the β-L-6dTal-1P reaction resulted in increased hydrolysis to form GMP as observed by comparison of t = 2 h (blue) and t = 6 h (red) reaction times by HPLC analysis.

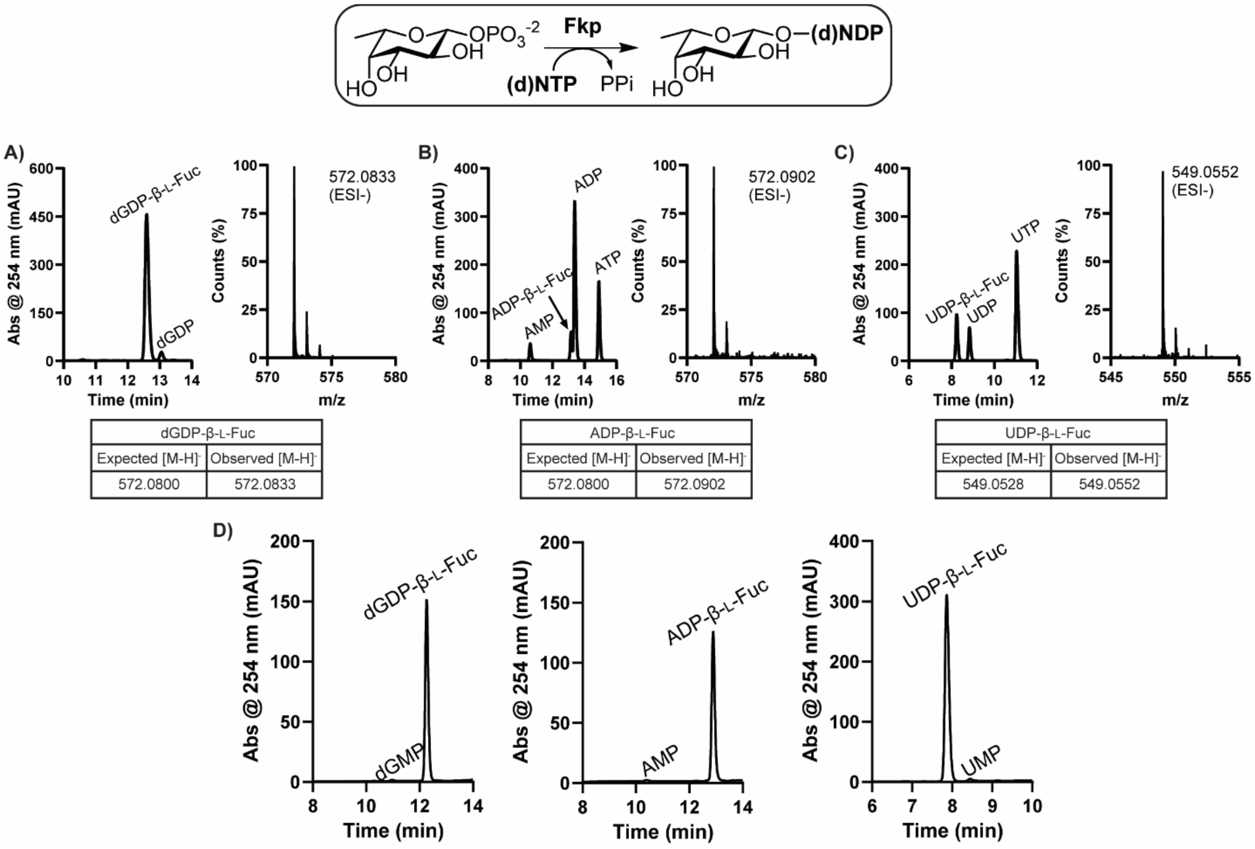

Figure S6. Characterization of each (d)NDP-β-L-Fuc produced by Fkp with different (d)NTPs using HPLC and HRMS. A) dGDP-β-L-Fuc, B) ADP-β-L-Fuc, and C) UDP-β-L-Fuc were produced from Fkp reactions containing 10 mM β-L-Fuc-1P and corresponding (d)NTPs (10 mM each). The production of (d)NDP-β-L-Fuc was confirmed by HPLC analysis (left) of reactions and HRMS analysis (right). D) HPLC analyses of above reactions following scale-up (1.5 mL) and purification by preparative HPLC.

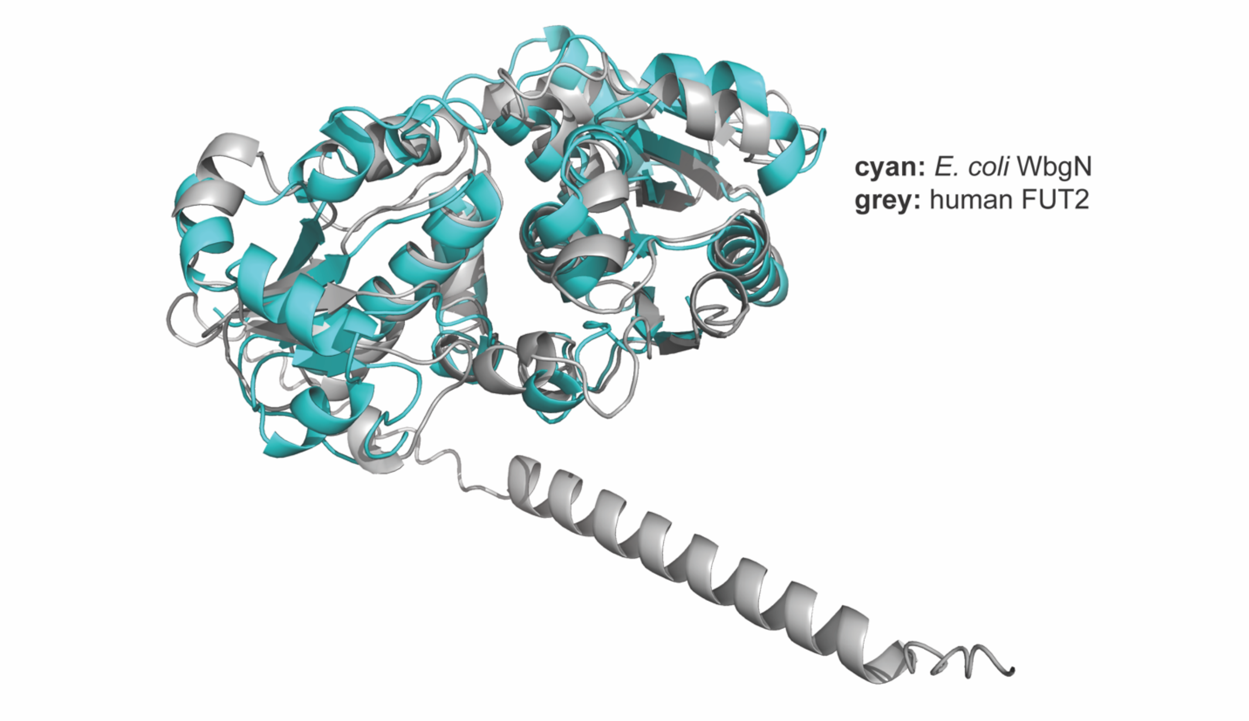

Figure S7. Structural alignment of *E. coli* WbgN and human FUT2 illustrates high structural similarity. AlphaFold structural models of *E. coli* WbgN and human FUT2 were aligned (superposed, sequence-independent, PyMol) with an RMSD of 1.624 Å, indicating high structural similarity.[7-9] FUT2 has an additional predicted transmembrane helix that is absent in *E. coli* WbgN.

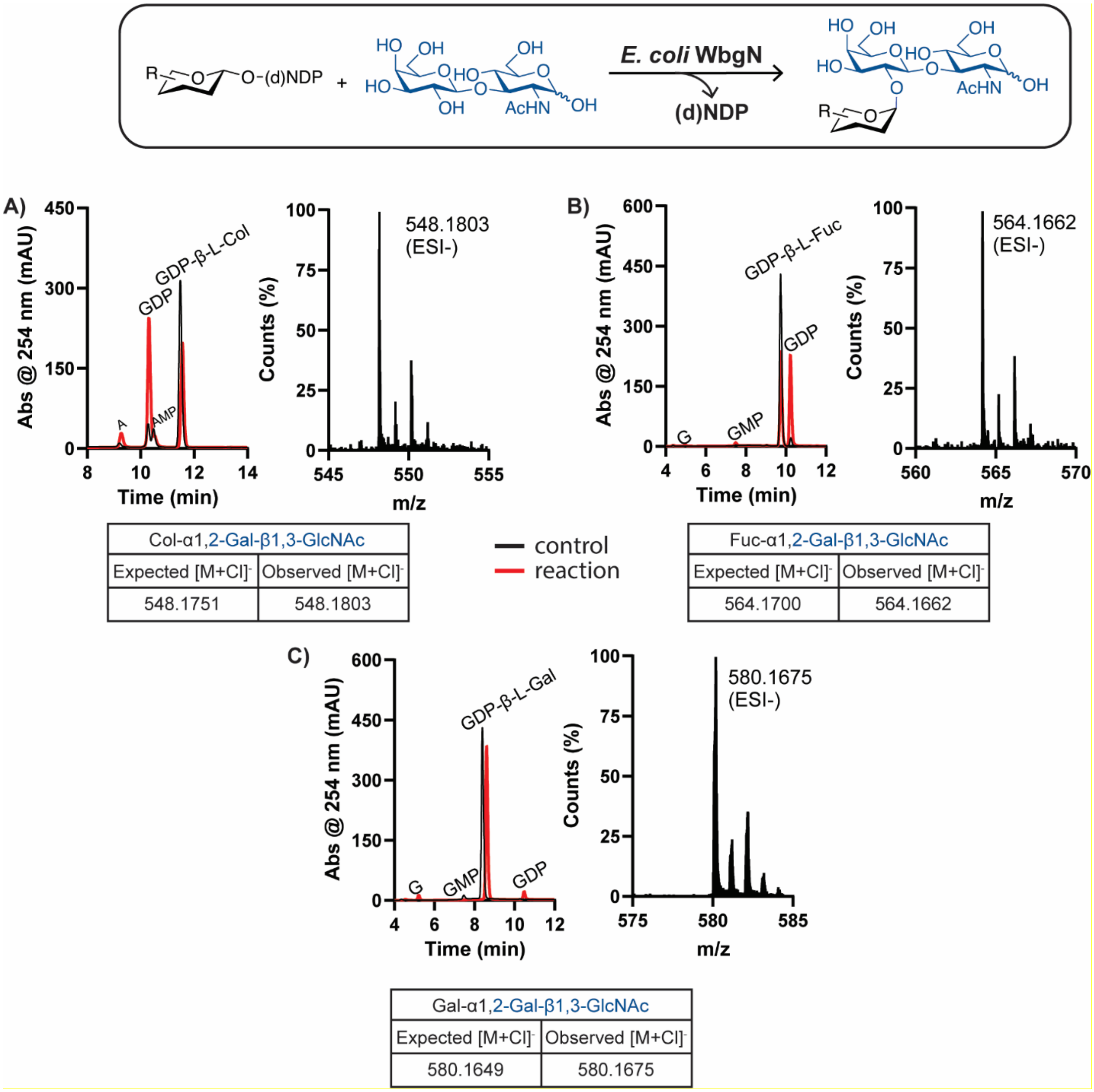

Figure S8. Characterization of WbgN reactions with lacto-*N*-biose (LNB) and NDP-β-L-sugars indicates production of trisaccharides. The production of trisaccharide products from 1 mM LNB and A) GDP-β-L-Col, B) GDP-β-L-Fuc, and C) GDP-β-L-Gal (1 mM each) reactions with 5 μM WbgN was indicated by HPLC analysis (left) and confirmed with HRMS analysis (right). The controls for each reaction were performed by substituting lacto-*N*-biose with water. In part C, the x-axis of the reaction was shifted by 0.2 min for better visualization of donor consumption.

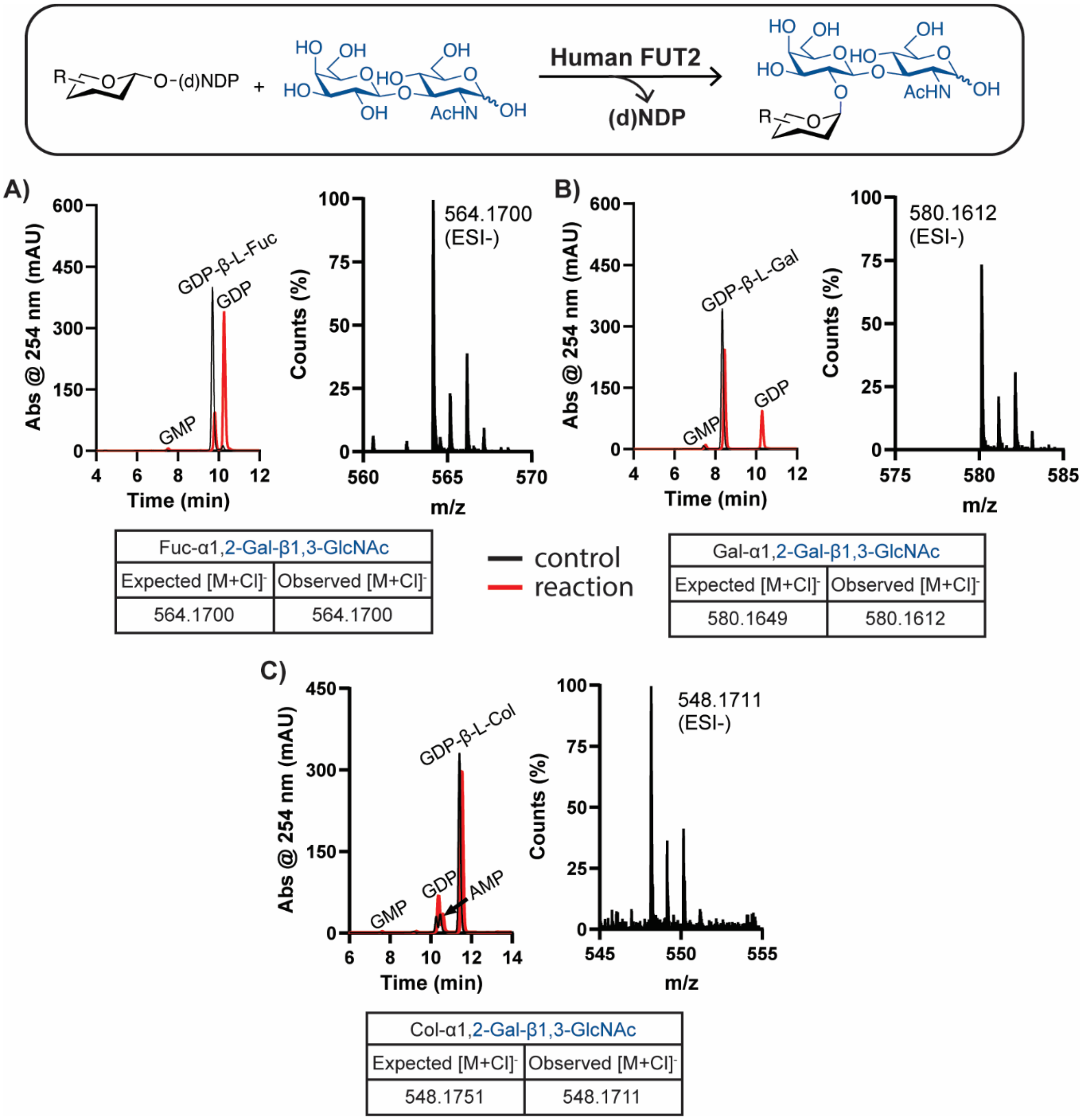

Figure S9. Characterization of FUT2 reactions with lacto-*N*-biose (LNB) and NDP-β-L-sugars indicates production of trisaccharides. A) L-Fuc, B) L-Gal, and C) L-Col were all transferred onto 1 mM LNB using 200 nM FUT2 and appropriate GDP-β-L-sugar donors (1 mM each) as indicated by HPLC analysis (left) and confirmed by HRMS analysis (right). The controls for each reaction were performed by substituting FUT2 with reaction buffer.

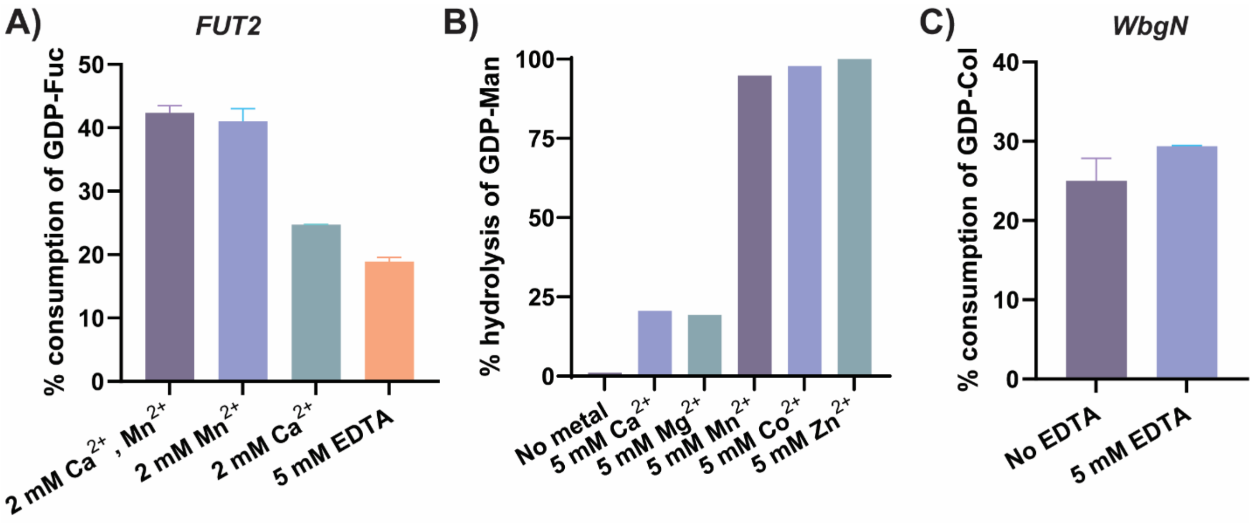

Figure S10. HPLC analysis of glycosyltransferase (GT) reactions indicates human FUT2 and *E. coli* WbgN exhibit different metal dependencies. A) Analysis of human FUT2 reactions with indicated metals or the metal chelator EDTA demonstrates that the addition of Mn^{2+} and/or Ca^{2+} promotes consumption of GDP-β-L-Fuc (1 mM) in the presence of 1 mM LNB in buffer (25 mM Tris, 150 mM NaCl, pH 7.5). B) Analysis of GDP-β-L-Man (1 mM) hydrolysis with indicated metals without addition of enzyme indicates the transition metals (Mn^{2+}, Co^{2+}, and Zn^{2+}) cause the hydrolysis of GDP-β-L-Man in the reaction buffer. Ca^{2+} was chosen as a supplement for FUT2 reactions moving forward because it activated GT reactions and promoted less hydrolysis of NDP-sugar in control reactions. C) Analysis of *E. coli* WbgN reactions with 1 mM GDP-β-L-Col and 1 mM LNB in the presence or absence of EDTA in buffer (5 mM Tris, 30 mM NaCl, pH 7.5) indicates that the WbgN reaction is not metal-dependent, as a metal scan was previously reported.[10] For parts A and C: Control reactions were performed for each GT reaction by substituting FUT2 with the reaction buffer, or by substituting LNB with water for the WbgN control reaction, and the hydrolysis of donor observed in the control reactions was subtracted from the donor consumption in each reaction. Bars indicate standard deviation (SD, n=2).

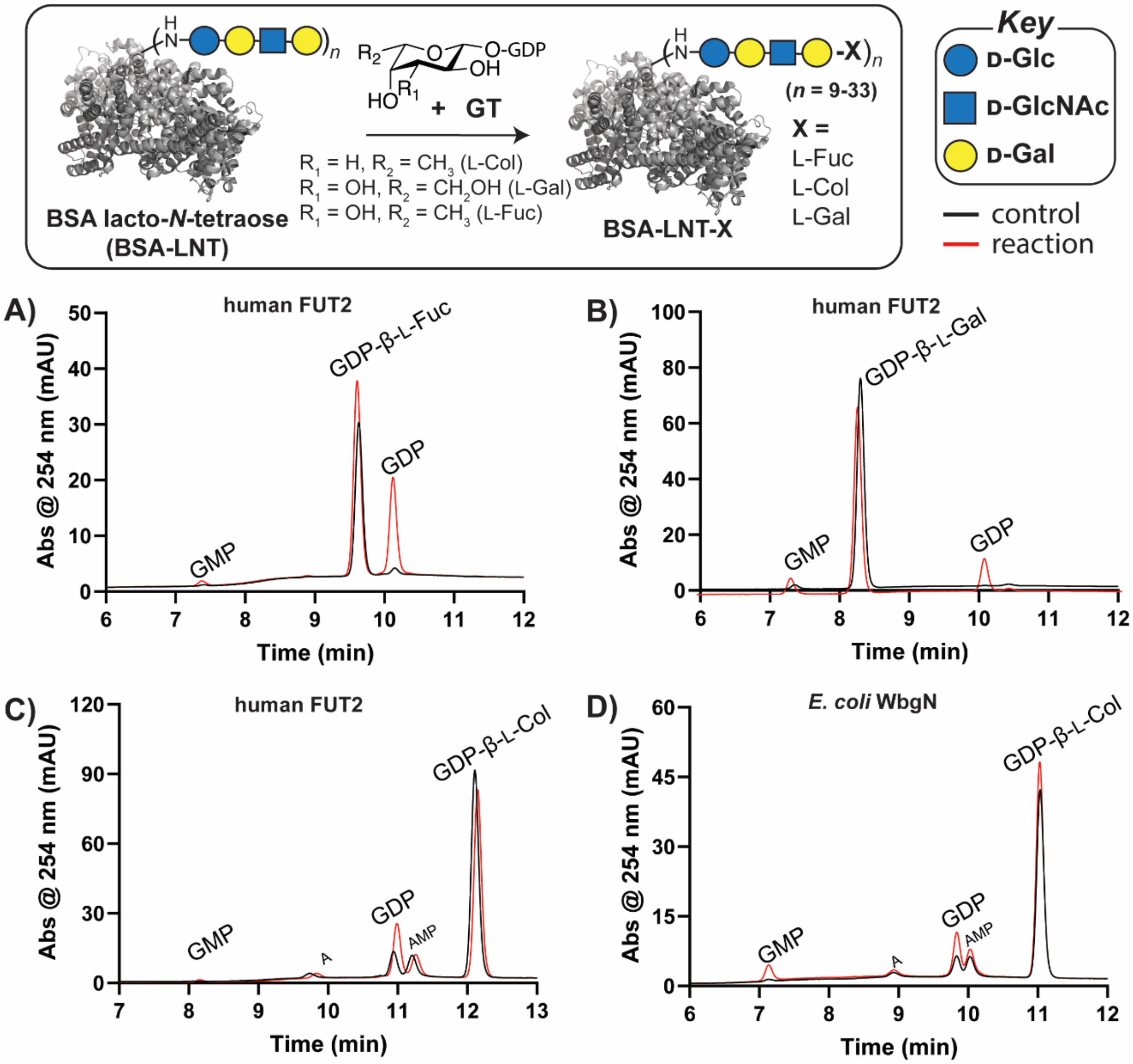

Figure S11. FUT2 and WbgN label glycoproteins with L-sugars. HPLC analysis indicates that BSA-lacto-*N*-tetraose (BSA-LNT, 0.5 mg/mL) is modified by FUT2 (50 nM, 200 nM and 360 nM, respectively) with A) L-Fuc, B) L-Gal, or C) L-Col with 600 µM GDP-β-L-Fuc, 700 µM GDP-β-L-Gal, or 1 mM GDP-β-L-Col used in each reaction, respectively (red lines). D) 10 µM WbgN labels BSA-LNT with L-Col using 1 mM GDP-β-L-Col as a donor. For each part, controls (black lines) were also performed by replacing the enzymes with reaction buffer.

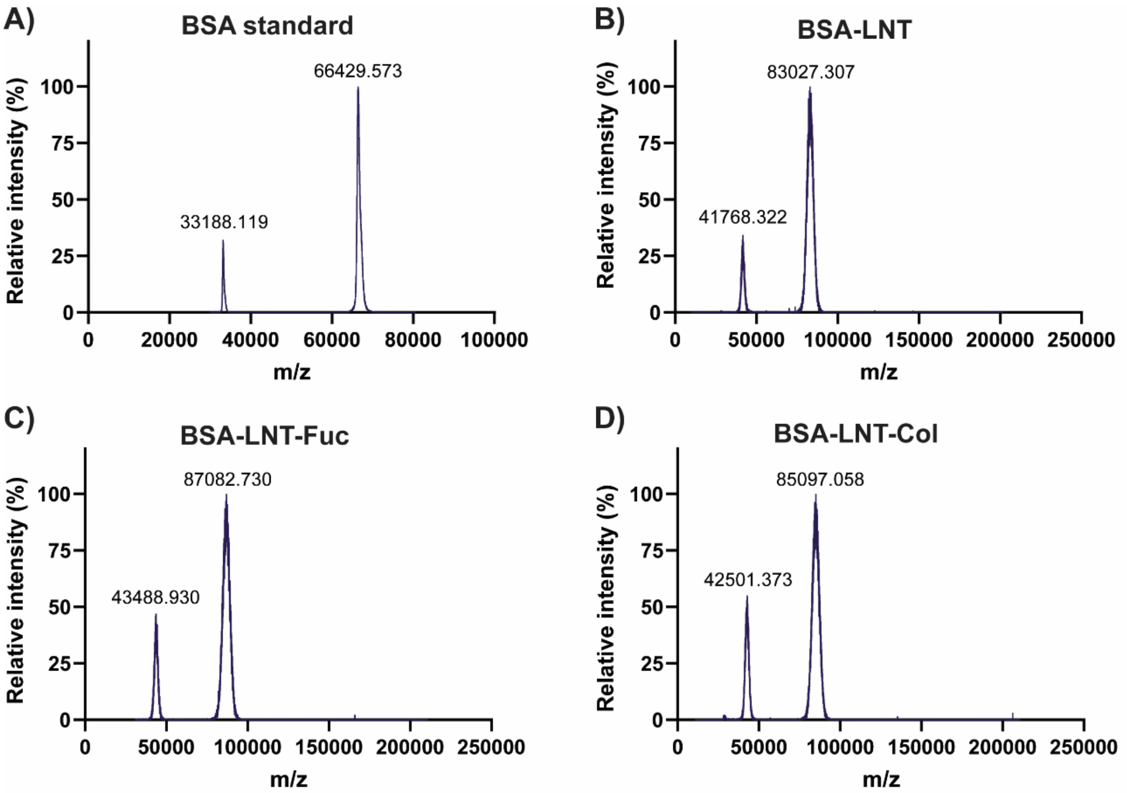

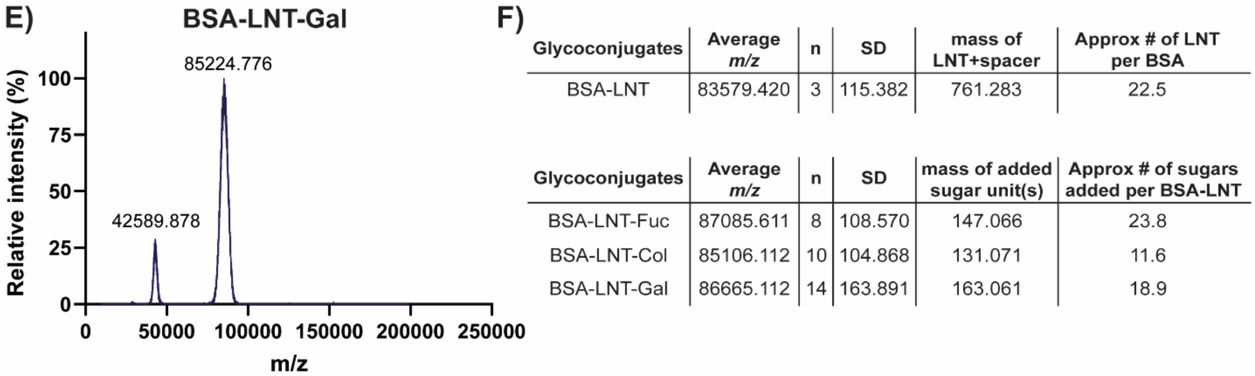

F)

Glycoconjugates	Average *m/z*	n	SD	mass of LNT+spacer	Approx # of LNT per BSA
BSA-LNT	83579.420	3	115.382	761.283	22.5

Glycoconjugates	Average *m/z*	n	SD	mass of added sugar unit(s)	Approx # of sugars added per BSA-LNT
BSA-LNT-Fuc	87085.611	8	108.570	147.066	23.8
BSA-LNT-Col	85106.112	10	104.868	131.071	11.6
BSA-LNT-Gal	86665.112	14	163.891	163.061	18.9

Figure S12. MALDI-TOF analysis of purified glycoproteins indicates attachment of L-sugars to BSA-LNT by FUT2. A) Analysis of calibrant Bovine Serum Albumin (BSA, expected mass 66430.3 Da).[11] B) Analysis of the substrate BSA-LNT. C-E) Representative MALDI-TOF traces of enzymatically labeled BSA-LNT-sugar conjugates used in this study following purification. Increase in m/z from that of part B indicates the addition of sugars onto BSA-LNT. F) Estimates of the number of LNT present per BSA (top, last column) or L-sugars added per BSA-LNT (bottom, last column) are indicated based on the molecular weight of each sugar unit and the observed m/z shift. The average m/z was calculated by averaging m/z values across n spectra of a given sample, as indicated in the table. 0.5 mg/mL of each glycoconjugate was loaded for analysis using super DHB as the matrix.

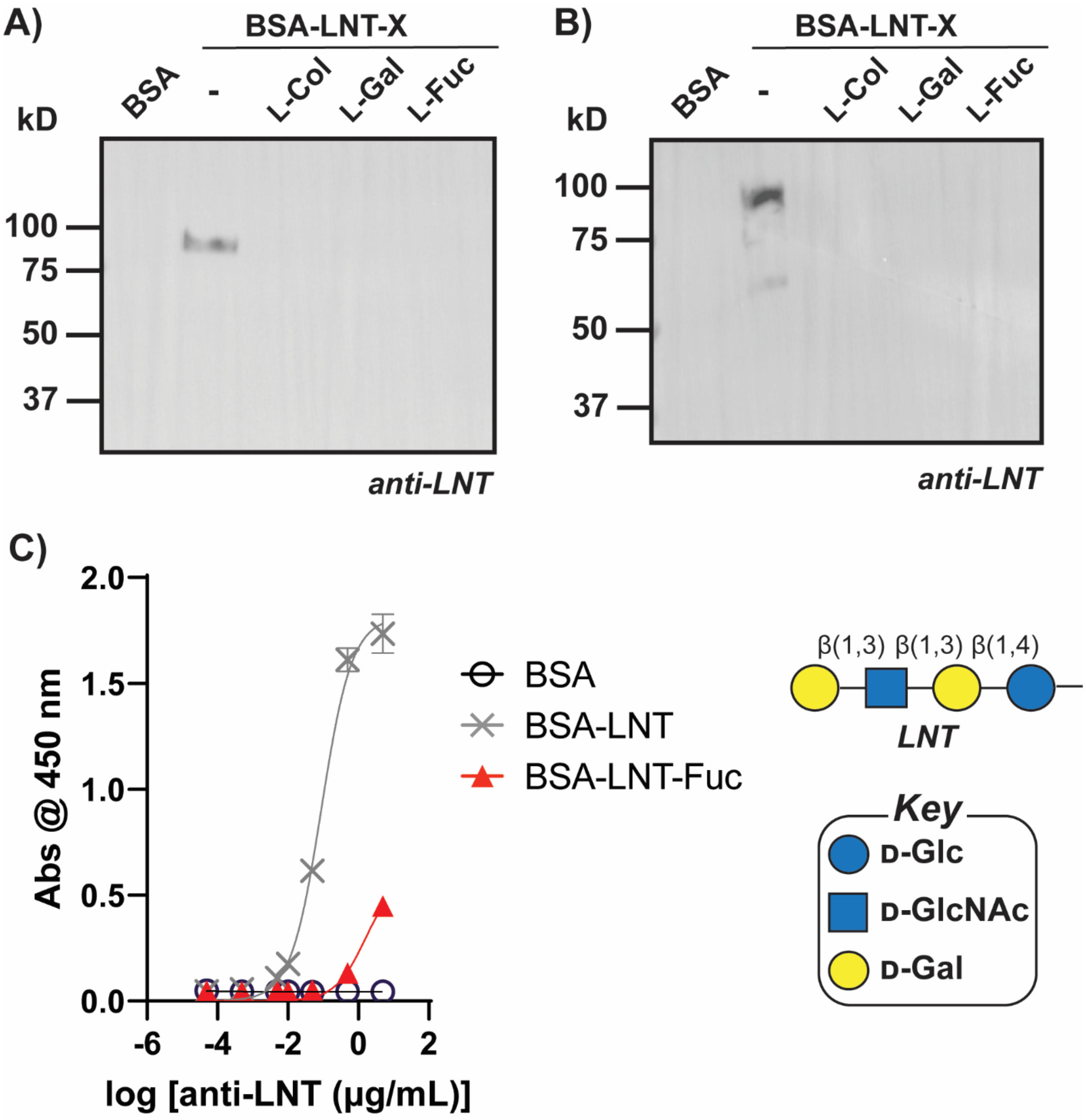

Figure S13. Anti-LNT primary antibody detects the starting material (BSA-LNT). A-B) Replicate experiments for western blot analysis shown in **Figure 4A**. C) Indirect ELISA analysis of indicated glycoproteins with an anti-LNT antibody indicates higher affinity binding to BSA-LNT than BSA-LNT-Fuc, as expected. Bars indicate SD (n = 3). No background subtraction was performed, as BSA control is shown. The native antigen sequence for anti-LNT is shown on the right.

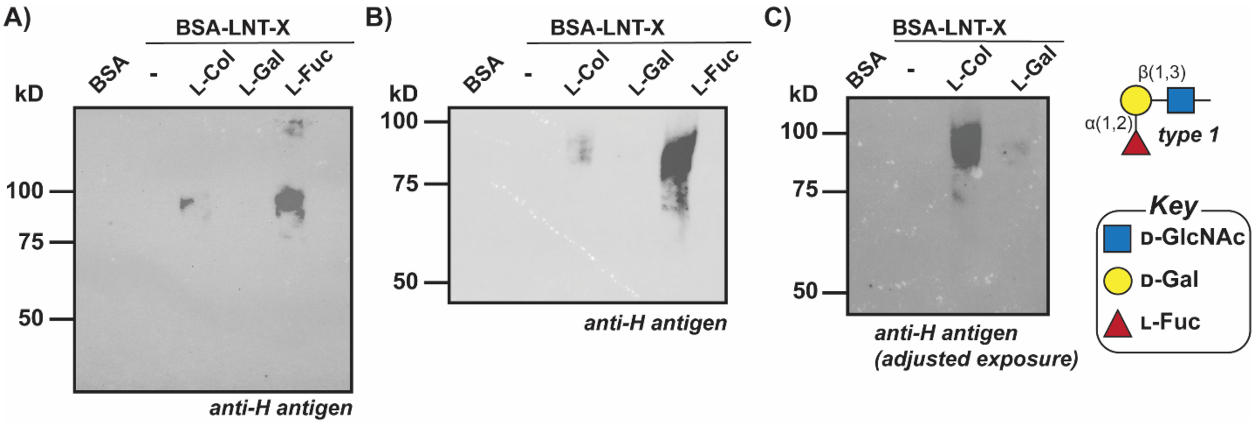

Figure S14. Anti-H antigen type 1 primary antibody recognizes glycoproteins containing terminal L-Fuc and L-Col residues. A-B) Replicate experiments for western blot analyses shown in **Figure 4B**. C) Western blot analysis with adjusted exposure for BSA-LNT-Col and BSA-LNT-Gal after removing BSA-LNT-Fuc sample from the blot in part B. The native antigen sequence for anti-H antigen (type 1) shown on the right.

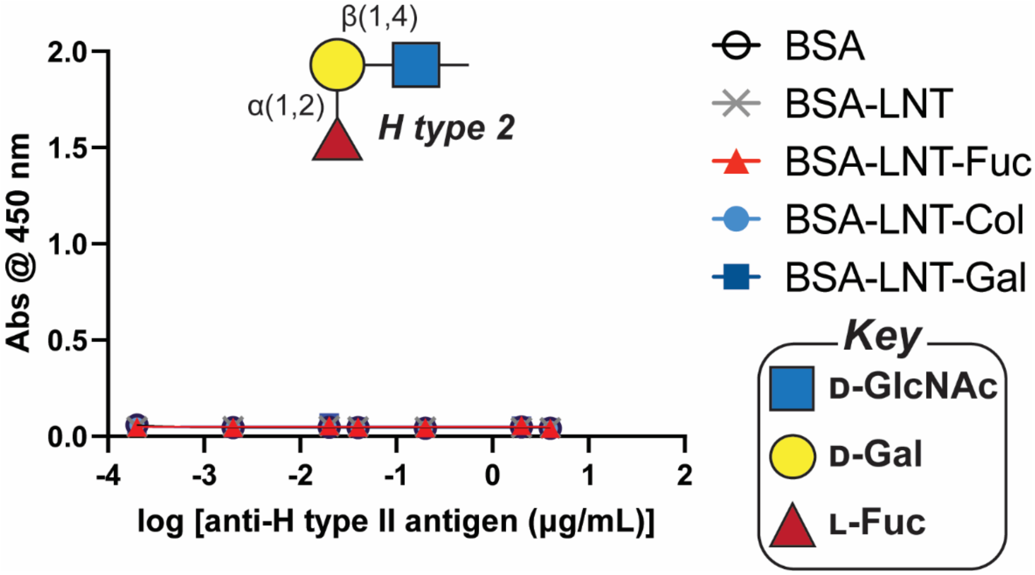

Figure S15. **Indirect ELISA analysis of semi-synthetic glycoproteins with anti-H antigen (type 2) primary antibody.** Using indicated antibody, no binding is detected for any of the glycoproteins. Bars indicate SD (n = 3). No background subtraction was performed, as BSA control is shown. The native antigen sequence for H-antigen (type 2) is shown on the top.

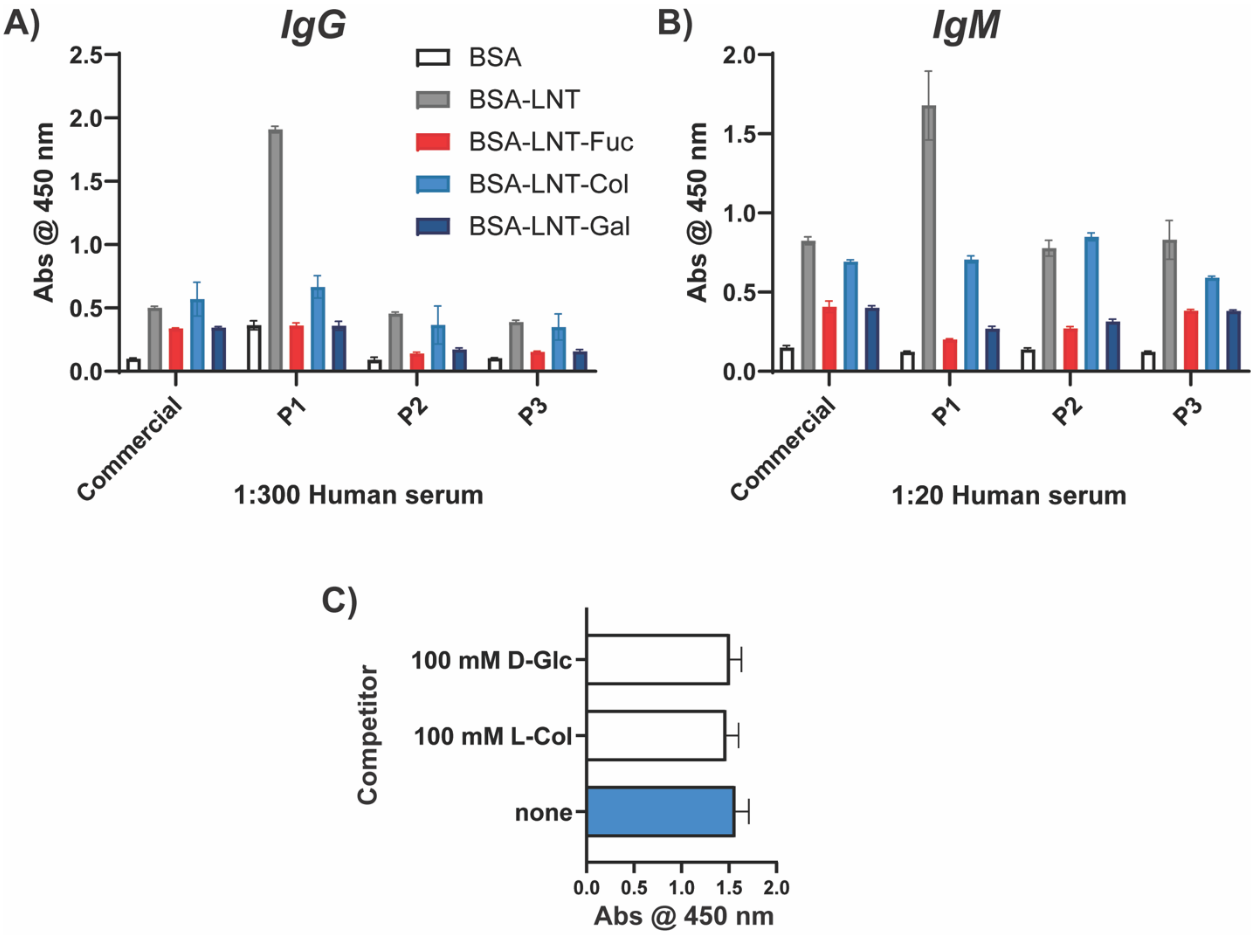

Figure S16. Indirect ELISA and competition analysis of engineered glycoproteins with commercial and clinical human serum samples using anti-human secondary antibodies. A-B) Indirect ELISA analysis of glycoproteins using commercial and clinical human serum samples as primary antibodies and A) anti-human IgG or B) anti-human IgM as secondary antibodies. A blank was set up for each experiment, where human serum is replaced by blocking buffer “B”, and the resulting absorbance values (typically < 0.05) were subtracted from the raw absorbance values for each. Bars indicate SD (n = 3). C) Competition ELISA analysis as shown in **Figure 4G** (bottom) for human IgA (P2) experiment with addition of free monosaccharides, as indicated. “None” indicates the competitor was replaced with an equal volume of water. Note that only a minor change in absorbance is observed upon addition of free sugars. Bars indicate SD (n=6).

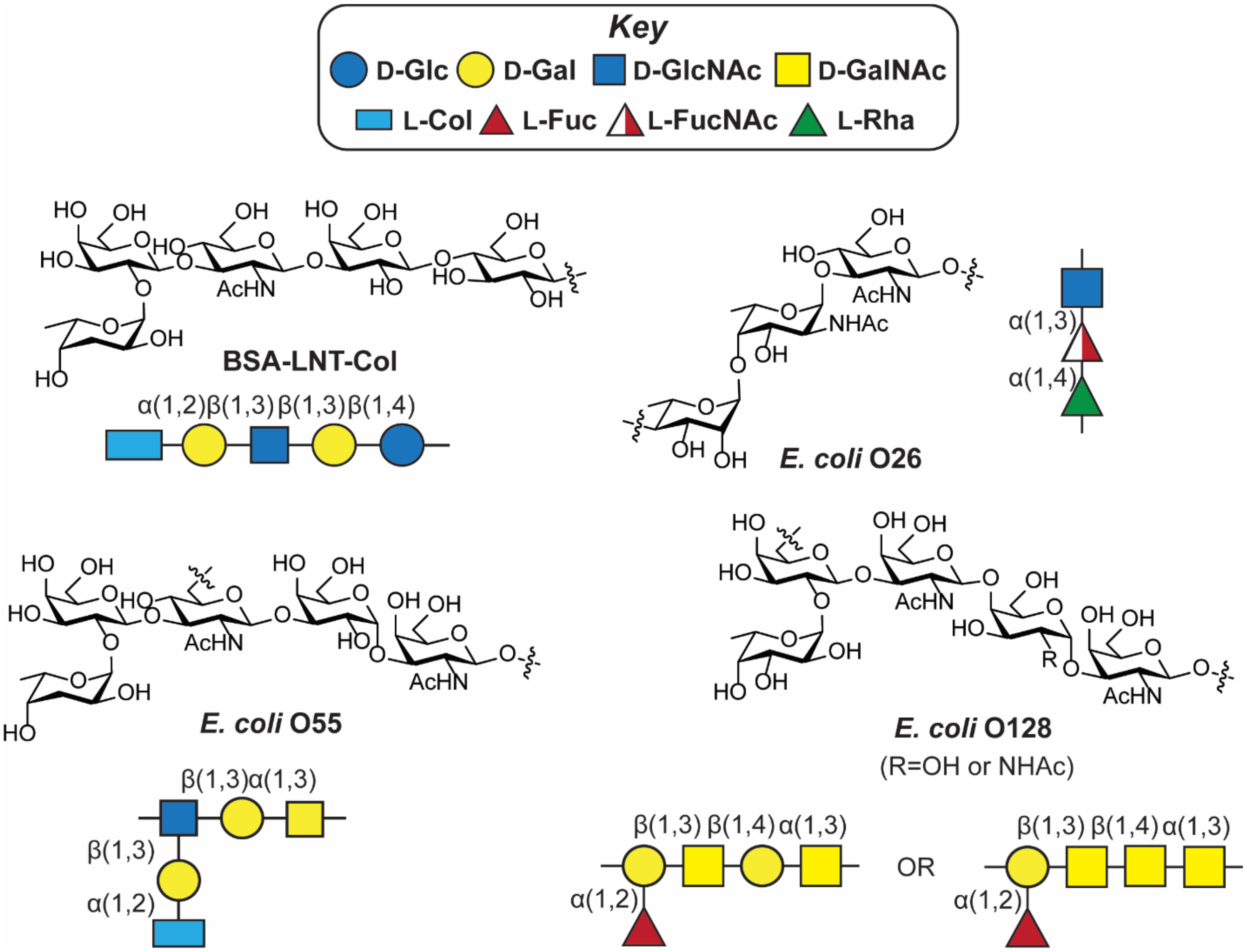

Figure S17. **Glycan structures of glycan component of BSA-LNT-Col and LPS O-unit repeat sequences used for competition assays in Figure 4G.** Note that variable structures exist for O128.

Table S1. NMR characterization and equilibrium ratios* of L-colitose isomers

L-Col isomer	Chemical shift (H-1, ppm)	Coupling constant ($^3J_{H-1,H-2}$, *Hz*)	Equilibrium ratio in D_2O
β-pyranose (**4p-β**)	4.54 (d)	8.0	11.1
α-pyranose (**4p-α**)	5.13 (d)	3.7	4.1
β-furanose (**4f-β**)	5.27 (s)	1.2	2.5
α-furanose (**4f-α**)	5.20 (d)	4.4	1

*Analyzed by ^{1}H NMR.

Table S2. Percent of indicated GDP-sugars produced* by Fkp over two steps (data shown in Figure 2D).

GDP-β-L-Fuc	GDP-β-L-Col	GDP-β-L-Gal	GDP-β-L-Glc
88.8 (3.6)	48.4 (6.6)	88.3 (10.1)	0.99 (0.03)

*Values in parentheses represent standard deviation (SD), n=3-9. Analyzed by HPLC.

Table S3. Percent of indicated GDP-sugars produced* from sugar-1-phosphates (data shown in Figure 2E).

time	GDP-β-L-Fuc	GDP-β-L-Rha	GDP-β-L-Man	GDP-β-L-6dTal
**2 h**	86.3 (4.3)	79.4 (5.4)	34.1 (4.3)	33.2 (4.3)
**6 h**	84.6 (2.0)	72.0 (5.1)	70.1 (7.1)	5.62 (0.89)

*Values in parentheses represent standard deviation (SD), n=6-7. Analyzed by HPLC.

Table S4. Percent of indicated NDP-β-L-sugars produced* by Fkp (data shown in Figure 2F).

GDP-β-L-Fuc	ADP-β-L-Fuc	CDP-β-L-Fuc	UDP-β-L-Fuc	dGDP-β-L-Fuc	dUDP-β-L-Fuc
93.8 (3.0)	10.0 (3.7)	0.8 (0.4)	34.8 (8.0)	82.0 (10.7)	0.1 (0.1)

*Values in parentheses represent standard deviation (SD), n=3-9. Analyzed by HPLC.

Table S5. Percent of donor consumption* by indicated GTs with 1 mM LNB as an acceptor (includes data shown in Figure 3A).

Donor	*E. coli* WbgN	Human FUT2
**GDP-β-L-Fuc**	47.1 (11.8)	66.5 (14.3)
**dGDP-β-L-Fuc**	16.7 (4.4)	43.8 (20.6)
**GDP-β-L-Gal**	11.3 (7.2)	24.2 (10.5)
**GDP-β-L-Rha**	0.977 (1.653)	N/A

**GDP-β-L-Man**	N/A	3.04 (4.57)
**UDP-β-L-Fuc**	2.57 (0.83)	0.702 (0.582)
**ADP-β-L-Fuc**	0.323 (0.510)	0.343 (0.661)
**GDP-β-L-Col**	47.3 (8.3)	9.42 (4.52)
**dTDP-β-L-Fuc**	N/A	0.133 (0.047)
**UDP-α-D-GlcNAc**	2.25 (0.19)	N/A

*Values in parentheses represent standard deviation (SD), n=3-8. Analyzed by HPLC. N/A indicates that donor consumption could not be measured under described conditions.

Table S6. Percent of donor consumption* by indicated GTs with various acceptor concentrations (data shown in Figure 3B).

**Acceptor concentration**		***E. coli* WbgN + 1 mM GDP-β-L-Col**	**Human FUT2 + 1 mM GDP-β-L-Fuc**
**Lacto-*N*-biose (LNB)**	**0.1 mM**	6.58 (1.47)	4.25 (0.75)
	**0.5 mM**	23.6 (2.7)	13.6 (1.3)
	**1 mM**	40.2 (1.8)	18.4 (4.8)
	**2 mM**	58.9 (1.0)	24.4 (4.7)
	**5 mM**	70.7 (0.8)	33.7 (5.7)
**Lacto-*N*-tetraose (LNT)****, ***	**0.1 mM**	7.88 (0.67)	5.40 (1.00)
	**0.5 mM**	23.9 (1.4)	12.2 (2.9)
	**1 mM**	41.8 (3.5)	16.4 (3.2)
	**2 mM**	61.8 (2.1)	20.4 (3.4)
	**5 mM**	71.7 (0.4)	26.2 (4.5)
**BSA-LNT**	**0.1 mg/mL**	3.53 (0.95)	17.7 (4.7)
	**0.5 mg/mL**	3.81 (1.01)	25.6 (4.5)
	**1 mg/mL**	4.28 (0.89)	34.1 (4.2)

*Values in parentheses represent standard deviation (SD), n=6. Analyzed by HPLC.

**Colα1-2Galβ1-3GlcNAcβ1-3Galβ1-4Glc (LNT-Col) HRMS m/z: $[M+Na]^+$ Calcd. For $C_{32}H_{55}NO_{24}Na$ 861.3040, Found 861.2966.

***Fucα1-2Galβ1-3GlcNAcβ1-3Galβ1-4Glc (LNT-Fuc) HRMS m/z: $[M+Na]^+[-H_2O]$ Calcd. For $C_{32}H_{53}NO_{24}Na$ 858.2850, Found 858.2821.

Table S7. Plasmids used in this study.

No.	Plasmid or Gene Name	Relevant Features	Notes	References
1	pET16b_*B. fragilis*_fkp	pET16b-N-term-His-*B. fragilis*-fkp	A gift from Laurie Comstock Lab (University of Chicago)	Coyne, M. J. et al., 2005[12]
2	pET27b	pET27b cloning vector C-terminal His tag	Novagen	N/A
3	*wbgN*	Linear gene fragment codon optimized for *E. coli* expression system with adaptors	Twist Bioscience #103211	N/A
4	pHK304	pET27b-C-term-His-*E. coli*-WbgN	Overlap extension PCR using pET27b and primers pET27b_*E. coli*_WbgN_FWD and pET27b_*E. coli*_WbgN_REV with *wbgN* gene as a template	This work

Table S8. Strains used in this study.

No.	Strain Name	Strain information	Source
1	BL21 (DE3)	*E. coli* cells engineered for high level protein expression	Novagen
2	EcpET16fkp	BL21 transformed with plasmid 1 (**Table S7**) for N-term-His *B. fragilis* Fkp overexpression	Coyne, M. J. et al., 2005[12]
3	EcHK304	BL21 transformed with plasmid 4 (**Table S7**) for C-term-His *E. coli* WbgN overexpression	This work

Table S9. Oligonucleotide primers used in this study.

Oligo Name	5'-3' sequence
pET27b_*E. coli* WbgN_FWD	ATGAAATACCTGCTGCCGACCGCTGCTGCTGGTCTGATGAGTATCGTGGTTGCACGCTTGG
pET27b_*E. coli* WbgN_REV	TTAGCAGCCGGATCTCAGTGGTGGTGGTGGTGGTGTTTTTCAATTCGAACCCATTTCTCTG

Table S10. Antibodies and lectin used in this study.

Product	Vendor/ Source	Catalog #	Additional information
Purified anti-Blood Group Antigen Precursor (BG1) Antibody	BioLegend	932102	Monoclonal mouse IgMκ antibody
Blood Group Antigen H (O) Type 1 Monoclonal Antibody (17-206)	Invitrogen	14-9810-82	Monoclonal mouse IgG3 antibody
ABO, Blood Group H Antigen Antibody (19-OLE)	Bio-Techne	NBP2-44628	Monoclonal mouse IgMκ antibody (H-antigen Type 2 specific)
Rabbit Anti-*E. coli* O55 LPS	SSI Diagnostica	85051(SS)	Polyclonal rabbit antiserum for *E. coli* O55
HRP Conjugated *Ulex europaeus* Lectin (Gorse, Furze) UEA-I	EY Laboratories	H-2201-1	HRP conjugated lectin
Goat Anti-Mouse IgM Antibody, μ chain, HRP conjugate	Sigma Aldrich	AP128P	HRP conjugated secondary antibody against the heavy chains of mouse IgM
HRP Goat anti-mouse IgG (minimal x-reactivity) antibody	BioLegend	405306	HRP conjugated secondary antibody against mouse IgG
HRP Donkey anti-rabbit IgG (minimal x-reactivity) Antibody	BioLegend	406401	HRP conjugated secondary antibody against rabbit IgG
Goat anti-Human IgG (H+L) Secondary Antibody, HRP	Thermo Fisher	A18805	HRP conjugated secondary antibody against human IgG
Anti-Human IgM (μ-chain specific)−Peroxidase antibody produced in goat	Sigma Aldrich	A0420	HRP conjugated secondary antibody against the heavy chains of human IgM
HRP Goat anti-human IgA Antibody	BioLegend	411002	HRP conjugated secondary antibody against the heavy chains of human IgA
Human serum	Sigma Aldrich	H4522	from human male AB plasma, USA origin, sterile-filtered
Human serum	NYU Langone Center for Biospecimen Research and Development	P.00251808<br>P.00055233<br>P.00018205	Serum acquired from three de-identified patients at NYU Langone Health

Supporting Experimental Methods

General information for biochemical assays. All reagents used for enzymatic reactions were purchased from Sigma-Aldrich, Chem-Impex International or Fisher Scientific, if not otherwise stated, without further purification. When appropriate, enzymatic reactions were monitored by thin layer chromatography on silica gel 60 F_{254} aluminum plates (developing solvent: isopropanol/ammonium acetate (1M)/acetic acid=7:3:1 (v/v/v)), and visualized under UV and/or by staining with ceric ammonium molybdate (CAM) followed by brief heating. All reactions were quenched with an equal volume of 0.05 % formic acid upon completion.

For analytical HPLC analysis, reactions were centrifuged briefly at 15,493 x g and 50 µL of each reaction was loaded onto a 96-well plate (Thermo Fisher) to be analyzed by a Thermo Scientific Dionex UltiMate 3000 UHPLC. A 15 µL sample was injected for analysis on a Gemini 5 µm NX C-18 column (150 x 4.6 mm, 110 Å, Phenomenex) with a linear gradient using 50 mM TEAB (triethylamine bicarbonate) and acetonitrile (ACN) (0%→10% ACN in TEAB) at a flow-rate of 1 mL/min for 23 min at 25 °C monitored at 254 nm. The HPLC spectra were processed by Chromeleon 7 software, and data plotting was performed using GraphPad Prism 9.0 software. 1 M TEAB buffer was prepared by diluting 139 mL of HPLC-grade triethylamine (TEA) into 700 mL of water and passing carbon dioxide from evaporated dry ice through the solution overnight.[13] The buffer was then diluted to 1 L to give 1 M TEAB stock solution that was stored at 4 °C. Before each use, the stock 1 M TEAB was diluted to 50 mM, and filtered.

HPLC purification of compounds was performed with a Thermo Scientific Dionex UltiMate 3000 UHPLC using a Gemini 5 µm NX C-18 preparative column (21.2 × 150 mm, 110 Å, Phenomenex) with a linear gradient using 50 mM TEAB and ACN (0%→10% ACN in TEAB) at a flow-rate of 5 mL/min for 56 min. Peaks detected at 254 nm were collected by an automated fractionation system. The relevant collected peak fractions were then flash-frozen in liquid N_2 and lyophilized. For MS analysis, each lyophilized sample was dissolved in 50 µL of HPLC grade water, transferred to a V-bottom vial (Thermo Fisher), and analyzed by an Agilent LC-TOF using ESI. For MALDI-TOF analysis of purified glycoconjugates, super-DHB (2,5-dihydroxybenzoic acid) matrix (50 mg/mL in 50% ACN/H_2O with 0.1 % trifluoroacetic acid (TFA)) was used as the spotting matrix for all samples. All glycoconjugates were spotted using the sandwich method with a 1:1 sample to matrix ratio. MS analysis was conducted using a Bruker UltraFlex MALDI-TOF in positive ion mode, and the laser power setting was adjusted for each sample. BSA (m/z = 66,430) was used as a calibrant.

Cloning of an expression vector for *E. coli* WbgN

All plasmids are listed in **Table S7**; strains are listed in **Table S8**; and primers are listed in **Table S9**. Overexpression plasmids (**Table S7**) were constructed using overlap extension PCR with indicated plasmid and primer pairs (**Tables S7 and S9**).[14] PCR products were DpnI (New England Biolabs) treated, purified using a PCR purification kit (Qiagen) and transformed into either Mach1 (Invitrogen) or DH5α competent cells (Novagen). Following confirmation by DNA sequencing (Genewiz), plasmids were transformed into BL21 (DE3) competent cells (Novagen) for overexpression.

Overexpression and Purification of *B. fragilis* Fkp

The N-terminal His_{10}-tagged Fkp plasmid (pET16b_*B. fragilis*_fkp in BL21, **Table S8**) was a gift from the Comstock lab (Harvard Medical School, now University of Chicago). Overexpression and purification were carried out following published protocols.[15]

Briefly, the Fkp plasmid was transformed into BL21 (DE3) competent cells, which was used to inoculate Luria-Bertani (LB) medium supplemented with 50 µg/mL carbenicillin on a shaking platform (200 RPM, 37 °C) overnight. The overnight culture (5 mL) was used to inoculate 500 mL of LB medium (1:100) supplemented with 100 µg/mL carbenicillin and incubated on a shaking platform until the OD_{600} (optical density at 600 nm) reached 1.2. Then, the culture was induced for protein

expression with 0.5 mM isopropyl-β-D-thiogalactopyranoside (IPTG) for 12 h (200 RPM, 21 °C). The induced cells were harvested by centrifugation (1857 x g, 20 min, 4 °C).

To purify the enzyme, the 250 mL pellet was resuspended in 25 mL of lysis buffer F (20 mM Tris(hydroxymethyl)aminomethane hydrochloride (Tris-HCl), pH 7.5, 500 mM NaCl, 10 % glycerol). The cells were sonicated (Fisherbrand, 5 min, 30 sec on/30 sec off, 50 % amplitude, twice with cooling between runs) on ice and the lysate was cleared by centrifugation (Beckman Coulter Allegra X-15R at 10,956 x g, 30 min, 4 °C). The supernatant was then loaded onto a Ni-NTA agarose resin (3 mL, Qiagen) column (pre-equilibrated with 2 column volumes (CVs) of lysis buffer F containing 500 mM imidazole followed by 4 CVs of lysis buffer F). After rocking the column at 4 °C for 30 min, the unbound proteins were washed off with 1 CV each of lysis buffer F containing 10, 30, 50 and 100 mM imidazole, and finally the target proteins were eluted with 1 CV of lysis buffer F containing 250 mM and then 500 mM imidazole. Fractions containing target protein were confirmed by SDS-PAGE. The eluted protein was then concentrated with a 30 kDa MWCO Amicon Ultra Centrifugal Filter Device (Millipore) and transferred into storage buffer (20 mM Tris-HCl, pH 7.5, 150 mM NaCl, 10 % glycerol) by centrifugation (1857 x g, 4 °C). The concentration of the pure protein was determined using the DC protein assay (Bio-Rad) using bovine serum albumin (BSA) as a standard. The protein was aliquoted, and flash-frozen in liquid N_2 to be stored at -80 °C until use.

Overexpression and Purification *E. coli* WbgN

Overexpression and purification were optimized, as a published protocol did not provide high yields of protein in our hands.[10] The *E. coli* WbgN plasmid was transformed into BL21 (DE3) competent cells and was used to inoculate LB medium supplemented with 25 µg/mL kanamycin, prior to shaking (200 RPM, 37 °C) overnight. The overnight culture (5 mL) was used to inoculate 500 mL of LB medium (1:100) supplemented with 25 µg/mL kanamycin, which was incubated on a shaking platform until the OD_{600} reached 0.6. Then, the culture was induced for protein expression with 0.1 mM IPTG for 12 h (200 RPM, 18 °C). The induced cells were harvested by centrifugation (1857 x g, 20 min, 4 °C).

For purification of WbgN, the 250 mL pellet was resuspended in 25 mL of lysis buffer W (20 mM Tris-HCl, pH 7.0, 300 mM NaCl, 10 % glycerol) supplemented with 0.2 % TritonX-100 and 0.06 mg/mL lysozyme. Cells were rocked for 30 min at 4 °C and were lysed by cell disruption (Avestin Emulsiflex C-5) on ice with a pressure of 10,000-15,000 PSI until clear. The lysate was cleared by centrifugation (Beckman Coulter Allegra X-15R at 10,956 x g, 30 min, 4 °C). The supernatant was then loaded onto a Co-NTA agarose resin (4 mL, Qiagen) column (pre-equilibrated with 2 CVs of the lysis buffer W containing 500 mM imidazole followed by 4 CVs of the lysis buffer W). After rocking the column at 4 °C for 30 min, the unbound proteins were washed off with 2 CVs each of lysis buffer W containing 5, 25, 50 and 100 mM imidazole, and finally the target proteins were eluted with 1 CV of lysis buffer W containing 250 mM imidazole. Fractions containing target protein were confirmed by SDS-PAGE. The eluted fractions were dialyzed overnight against 1 L of dialysis buffer (20 mM Tris-HCl, pH 7.0, 300 mM NaCl, 30% glycerol, 2 mM dithiothreitol (DTT)) at 4 °C in a 10 kDa MWCO Slide-A-Lyzer dialysis cassette (Thermo Scientific). The dialyzed fractions are then concentrated in the 10 kDa MWCO Amicon Ultra Centrifugal Filter Device (Millipore) by centrifugation (1857 x g, 4 °C). The concentration of the pure protein was reported using the DC protein assay (Bio-Rad) supplemented with reagent S, using BSA as a standard. The protein was aliquoted, and flash-frozen in liquid N_2 to be stored at -80 °C until use.

Fkp two-step reaction for the production of GDP-sugars from monosaccharides

To examine the substrate scope of Fkp, a 24 µL reaction was set up containing 10 mM L/D-sugar, 10 mM ATP and 16 µg (5 µM) of purified Fkp in reaction buffer F (100 mM Tris-HCl, pH 8.0, 20 mM $MgCl_2$) and the reaction was allowed to proceed for t = 5 h at 25 °C. The production of sugar-1Ps was monitored by TLC. Then, 10 mM GTP and 8 µg/mL inorganic pyrophosphatase were added to the above reactions (now a total volume of 30 µL) and the reaction was allowed to proceed for t = 2 h or

18 h. The reaction was then treated with 1 U of apyrase, which converted the remaining nucleotides to AMP and GMP, at each time point for t = 1 h at 37 °C and quenched. The formation of GDP-sugars was analyzed by HPLC and HRMS.

Fkp nucleotidyltransferase activity assays

To test the substrate promiscuity of Fkp with various β-L-sugar-1Ps, 30 µL reactions were set up containing 10 mM sugar-1Ps, 10 mM GTP, 8 µg/mL inorganic pyrophosphatase and 16 µg (5 µM) of purified Fkp in the reaction buffer F, and the reaction was allowed to proceed for t = 2 h or 6 h at 25 °C. The reaction was then quenched at each time point, and the formation of GDP-sugars was assessed by HPLC and HRMS.

To test the substrate promiscuity of Fkp with various nucleotides, 30 µL reactions were set up containing 10 mM β-L-Fuc-1P, 10 mM (d)NTPs, 8 µg/mL inorganic pyrophosphatase and 16 µg (5 µM) of purified Fkp in the reaction buffer F, and the reaction was allowed to proceed for t = 18 h at 25 °C. The reaction was then quenched, and the formation of (d)NDP-β-L-Fuc was assessed by HPLC and HRMS.

***E. coli* WbgN/Human FUT2 substrate scope assays**

To test the substrate scopes of *E. coli* WbgN towards various NDP-sugar donors, 20 µL reactions were set up containing 1 mM (d)NDP-β-L-sugars, 1 mM lacto-*N*-biose and 3.5 µg (5 µM) of purified WbgN in reaction buffer W (5 mM Tris, pH 7.5, 30 mM NaCl, 5 mM EDTA). Controls for each NDP-sugar were carried out in which lacto-*N*-biose was replaced by an equal volume of water. Both controls and reactions were allowed to proceed for t = 18 h at 37 °C. The reactions and controls were then quenched and subjected to HPLC analysis. The consumption of NDP-sugars was quantified by integration of NDP-sugar donor peaks relative to the total amount of nucleotide-containing peaks. Percent donor remaining in each appropriate control was then subtracted from those of each reaction.

The substrate scope of human FUT2 (BioTechne) towards various NDP-sugars was carried out and analyzed as described above for WbgN, except 0.144 µg (200 nM) of human FUT2 and reaction buffer FT (25 mM Tris, pH 7.5, 150 mM NaCl, 2 mM $CaCl_2$) was used. Production of the trisaccharide products by WbgN and FUT2 was confirmed by HRMS.

To test the substrate scopes of *E. coli* WbgN and human FUT2 towards various acceptors, 20 µL reactions were set up containing 1 mM GDP-β-L-Col or Fuc, respectively, and lacto-*N*-biose, lacto-*N*-tetraose (0.1, 0.5, 1, and 5 mM), or lacto-*N*-tetraose-BSA (BSA-LNT, 0.1, 0.5, and 1 mg/mL) as an acceptor. The reactions were initiated by adding 3.5 µg (5 µM) of purified WbgN or 72 ng (100 nM) FUT2 in their respective reaction buffers and analyzed as described above. BSA-LNT was purchased from Dextra Laboratories and used without further purification for enzymatic reactions.

Synthesis and purification of glycoconjugates

For the fucosylation of BSA-LNT, two 75 µL reactions were set up containing 0.5 mg/mL BSA-LNT, 600 µM GDP-β-L-Fuc, and 36 ng (50 nM) of human FUT2 in reaction buffer FT. For transfer of L-Col and L-Gal, 700 µM GDP-β-L-Gal and 1 mM GDP-β-L-Col were used with 0.144 µg (200 nM) and 0.260 µg (360 nM) of human FUT2, respectively. Control reactions were also set up for each GDP-sugar by replacing human FUT2 with reaction buffer FT. Both controls and reactions were allowed to proceed for t = 18 h at 37 °C. The reaction progress was monitored by quenching 5 µL of both control and FUT2 reactions and analyzing donor usage by HPLC. The consumption of GDP-sugars was quantified relative to the controls as described above. The number of each sugar added was estimated by a combination of HPLC and MALDI-TOF analysis.

For purification of the glycoconjugates to remove His-tagged FUT2, two 70 µL reactions were combined and added to a pre-equilibrated Ni-NTA column (200 uL Ni-NTA resin). The column was washed with 5 mM Tris, pH 8.0 (8 x 1 mL), and all the flow-through fractions containing BSA conjugates were combined and concentrated using 50 kDa MWCO Pierce™ Protein Concentrators

(Thermo Scientific) by centrifugation (15,493 x g, 4 °C). The concentration of the glycoconjugates was determined by measuring the absorbance of the BSA conjugate by Nano Drop (Thermo Scientific) at 280 nm and using the molar extinction coefficient of BSA.

Gel electrophoresis and immunoblot analysis of glycoconjugates

For SDS-PAGE/Coomassie blue analysis, 1 µg of BSA, BSA-LNT, and each of the purified glycoconjugates were boiled at 100 °C for 10 min, loaded on a 9 % polyacrylamide Tris-Glycine gel, and analyzed by SDS-PAGE (180 V for 50 min using Tris-Glycine SDS running buffer). The gel was then stained with Coomassie blue stain and destained for imaging.

For immunoblot analysis, 0.5 µg of samples were analyzed by SDS-PAGE as described above. Protein conjugates were transferred onto polyvinylidene difluoride membrane (PVDF) at 25 V, 10 A for 30 min prior to incubation with blocking buffer M (2.5% non-fat milk in 1X TBS (tris-buffered saline) supplemented with 0.1 % Tween-20 (TBS-T)) for 1 h at 25 °C on a shaking platform. Primary antibody, mouse anti-human blood group antigen H (O) Type 1 IgG3 monoclonal antibody (mAb) (Invitrogen, 1:1K) or mouse anti-human blood group antigen precursor IgM mAb (Biolegend, 1:10K), in blocking buffer M was added and incubated overnight at 4 °C on a shaking platform. After washing with TBS-T for 2 h at 25 °C, the membrane was incubated with the secondary antibody, goat anti-mouse IgG HRP (1:10K, BioLegend) or goat anti-mouse IgM HRP (1:50K, Sigma Aldrich) respectively, in blocking buffer M for 1.5 h at 25 °C. Final washing with TBS-T was performed for 2 h at 25 °C with rocking prior to imaging with Clarity Max™ Western ECL Substrate (Bio-Rad).

Enzyme-linked immunosorbent assay (ELISA) of glycoconjugates with various antibodies

For analysis of Anti-H antigen antibodies with the purified glycoconjugates, multiwell plates (Pierce™ 96-well polystyrene plates, Thermo Fisher) were coated with 50 µL of 2 µg/mL of BSA, BSA-LNT, and BSA-LNT-Fuc/Col/Gal (0.1 µg per well) in 50 mM $NaHCO_3$, pH 9.2 (coating buffer) overnight at 4 °C. After washing (3 x 200 µL 1X TBS-T, 5 min each), the wells were blocked with 200 µL of blocking buffer B (2 % BSA in TBS-T) for 1.5 h on a shaking platform at 25 °C. Then, the primary antibody, mouse anti-human blood group antigen H (O) Type 1 IgG3 mAb (Invitrogen) or mouse anti-human blood group antigen H (O) Type 2 IgM mAb (Bio-Techne), was added at 8 different dilutions (starting with 10 or 4 µg/mL to 0, respectively) in blocking buffer B; each well was incubated with 50 µL of diluted primary antibodies or blocking buffer B for blank at 25 °C for 1.5 h. After washing the wells (3 x 200 µL TBS-T, 5 min each), 50 µL of secondary antibody, goat anti-mouse IgG HRP (1:10K, BioLegend) or goat anti-mouse IgM HRP (1:50K, Sigma Aldrich) respectively, in blocking buffer B was incubated for 1.5 h at 25 °C. After washing the wells (6 x 200 µL TBS-T, 5 min each), 50 µL of tetramethylbenzidine (TMB, Thermo Fisher) was added to each well and incubated at 25 °C for 4 min or 30 min respectively, before quenching with 50 µL of 2 M sulfuric acid. Absorbance of each well was monitored at 450 nm using a plate reader (SpectraMax iD5, Molecular Devices) prior to analysis in SoftMax Pro 7.1 and Graphpad PRISM. The absorbance was plotted against the log of the concentration of each antibody and the data was analyzed using following equation (where F is set to 50 to measure the EC50):

logEC50=logECF - (1/HillSlope)*log(F/(100-F))
Y=Bottom + (Top-Bottom)/(1+10^((LogEC50-X)*HillSlope))

For ELISA analysis using Anti-LNT antibody with glycoconjugates, the plates were coated with 50 µL of 10 µg/mL BSA, BSA-LNT, and BSA-LNT-Fuc (0.5 µg per well) as described above. Mouse anti-human blood group antigen precursor IgM mAb (5 to 0 µg/mL over a dilution series, BioLegend) was used as the primary antibody and goat anti-mouse IgM HRP (1:50K, Sigma Aldrich) was used as the secondary antibody. TMB was incubated at 25 °C for 3 min before quenching, and absorbance was measured at 450 nm. PRISM analysis was performed as above.

For ELISA analysis using Anti-O55 antibody with glycoconjugates, the plates were coated with 50 µL of 2 µg/mL of BSA, BSA-LNT, or BSA-LNT-Fuc/Col/Gal (0.1 µg per well) as described above. Rabbit anti-O55 sera polyclonal Ab (1:10, 1:50, 1:100, 1:200, 1:400, 1:1K, 1:2K, and 0, SSI Diagnostica) was used as the primary antibody and goat anti-rabbit IgG HRP (1:3K, BioLegend) was used as the secondary antibody. TMB was incubated at 25 °C for 9 min before quenching, and absorbance was measured at 450 nm. PRISM analysis was performed as above. For competition ELISA assays with LPS, anti-O55 (1:100) in blocking buffer B was incubated with 5 µL of indicated concentrations of each LPS (0.5, 1, 2.5, or 5 µg per well) in a total volume of 50 µL at 25 °C for 20 min and added into the wells coated with BSA-LNT-Col after the blocking step. For control wells (BSA-LNT-Col without competitor or "none"), an equal volume of water was added in place of LPS.

For ELISA analysis of human serum with glycoconjugates, the plates were coated with 50 µL of 10 µg/mL of BSA, BSA-LNT, and BSA-LNT-Fuc/Col/Gal (0.5 µg per well) as described above. The wells were blocked with blocking buffer B for 2 h on a shaking platform at 25 °C. Both clinical and commercial human serum samples were diluted to 1:300 for Anti-IgG experiments or 1:20 for both Anti-IgM and Anti-IgA experiments in blocking buffer B and incubated in each well for 2 h at 25 °C. Goat anti-human IgG HRP (1:10K, Thermo Fisher), goat anti-human IgM HRP (1:3K, Sigma Aldrich), or goat anti-human IgA HRP (1:3K, BioLegend), were used as secondary antibodies in separate experiments. Wash steps were performed as described above. TMB was incubated at 25 °C in each well for 20 min before quenching, and absorbance was measured at 450 nm. PRISM analysis was performed as above. For competition ELISA assays, human serum sample P2 (P.00055233, 1:20 dilution in blocking buffer B) was incubated in the wells coated with BSA-LNT-Col for 1 h at 25 °C as described above. After washing the wells (1 x 200 µL TBS-T), 5 µL of various concentrations of each competitor (5 or 50 µg LPS, 5 µg BSA-LNT or BSA-LNT-Col, or 100 mM L-Col or D-Glc) in a total volume of 50 µL TBS-T were added to each well and were incubated for additional 1 h at 25 °C on a shaking platform. For control wells ("none"), an equal volume of water was added in place of the competitor. Goat anti-human IgA HRP (1:3K) was used as a secondary antibody.

For direct lectin-based ELISA assays using UEA-I-HRP with glycoconjugates, the plates were coated with 50 µL of 10 µg/mL BSA, BSA-LNT, and BSA-LNT-Fuc/Col/Gal (0.5 µg per well) as described above. After washing the wells (3 x 200 µL 1X phosphate buffer saline (PBS) supplemented with 0.2 % Tween-20 (PBS-T), 5 min each), the wells were blocked with 200 µL of blocking buffer C (1X Carbo-free blocking solution (Vector Laboratories) in water supplemented with 0.2 % Tween-20) for 2 h on a shaking platform at 25 °C. Then, the HRP conjugated UEA-I was examined at 5 different dilutions (100 to 0 µg/mL) in blocking buffer C supplemented with 1 mM $MnCl_2$ and 1 mM $CaCl_2$; each well was incubated with 50 µL of diluted UEA-I-HRP or blocking buffer C with supplemented metal ions as the blank at 25 °C for 2 h. For the wells without metal, UEA-I-HRP was diluted in blocking buffer C without addition of any metal. After washing the wells (6 x 200 µL PBS-T, 5 min each), TMB was incubated at 25 °C for 20 min before quenching and absorbance was measured at 450 nm. PRISM analysis was performed as above.

NMR of Benzyl-L-fucopyranoside (1)

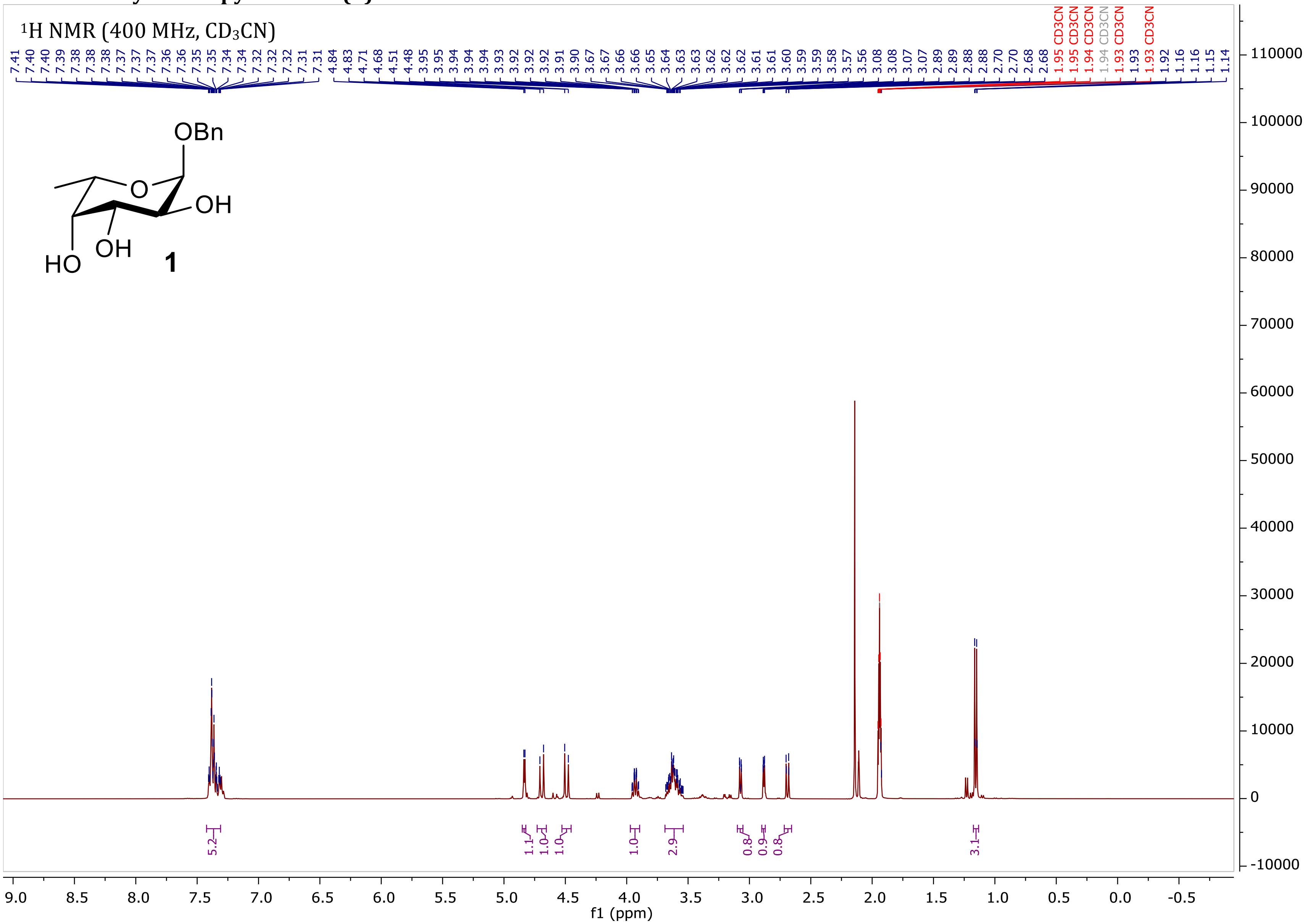

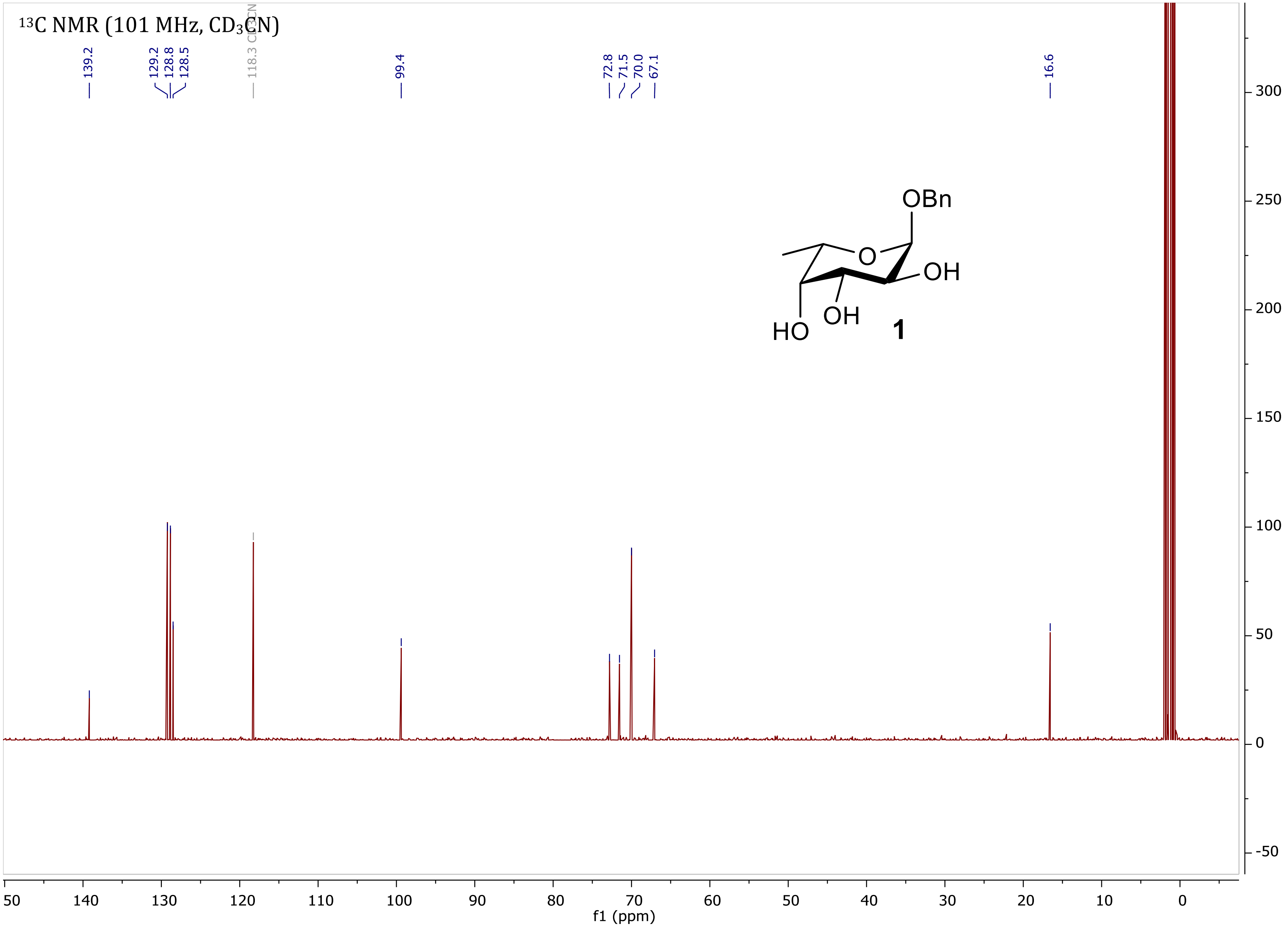

13C NMR (101 MHz, CD3CN)
139.2
129.2
128.8
128.5
118.3 CD3CN
99.4
72.8
71.5
70.0
67.1
16.6
OBn
O
OH
OH
HO
1
f1 (ppm)

DEPT-135 NMR (101 MHz, CD_3CN)

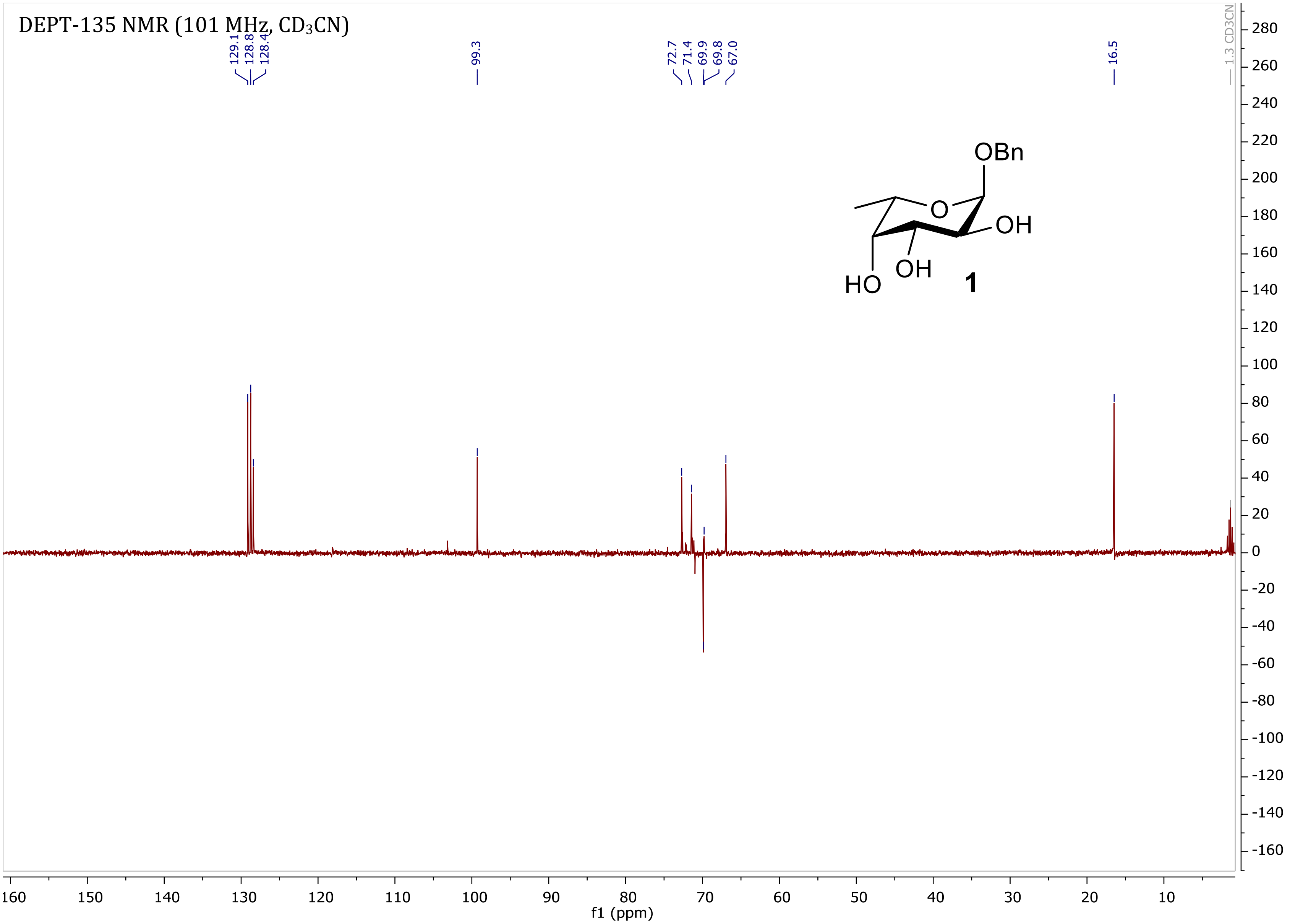

NMR of Benzyl 3-*O*-phenoxythiocarbonyl-α-L-fucopyranoside (2)

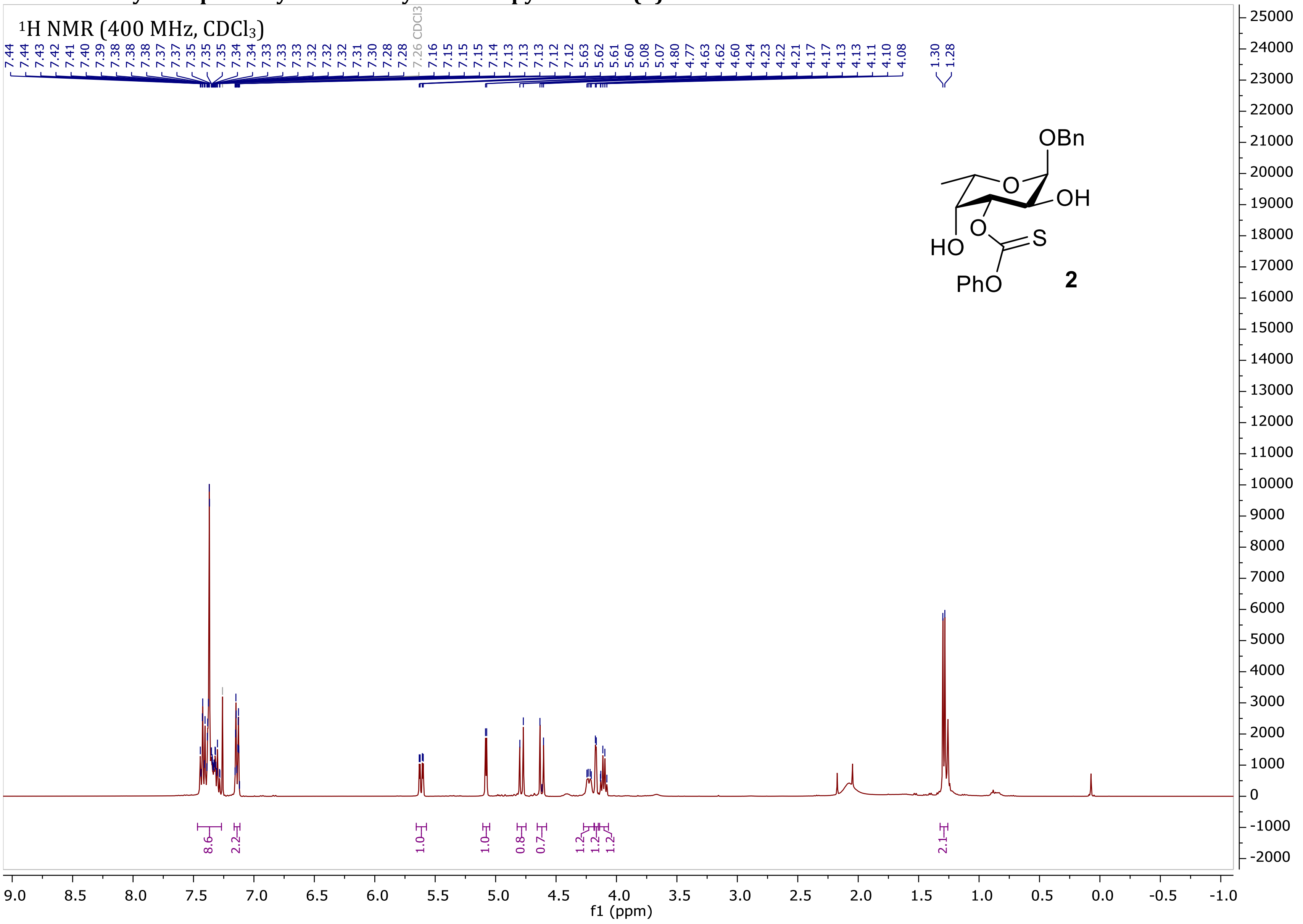

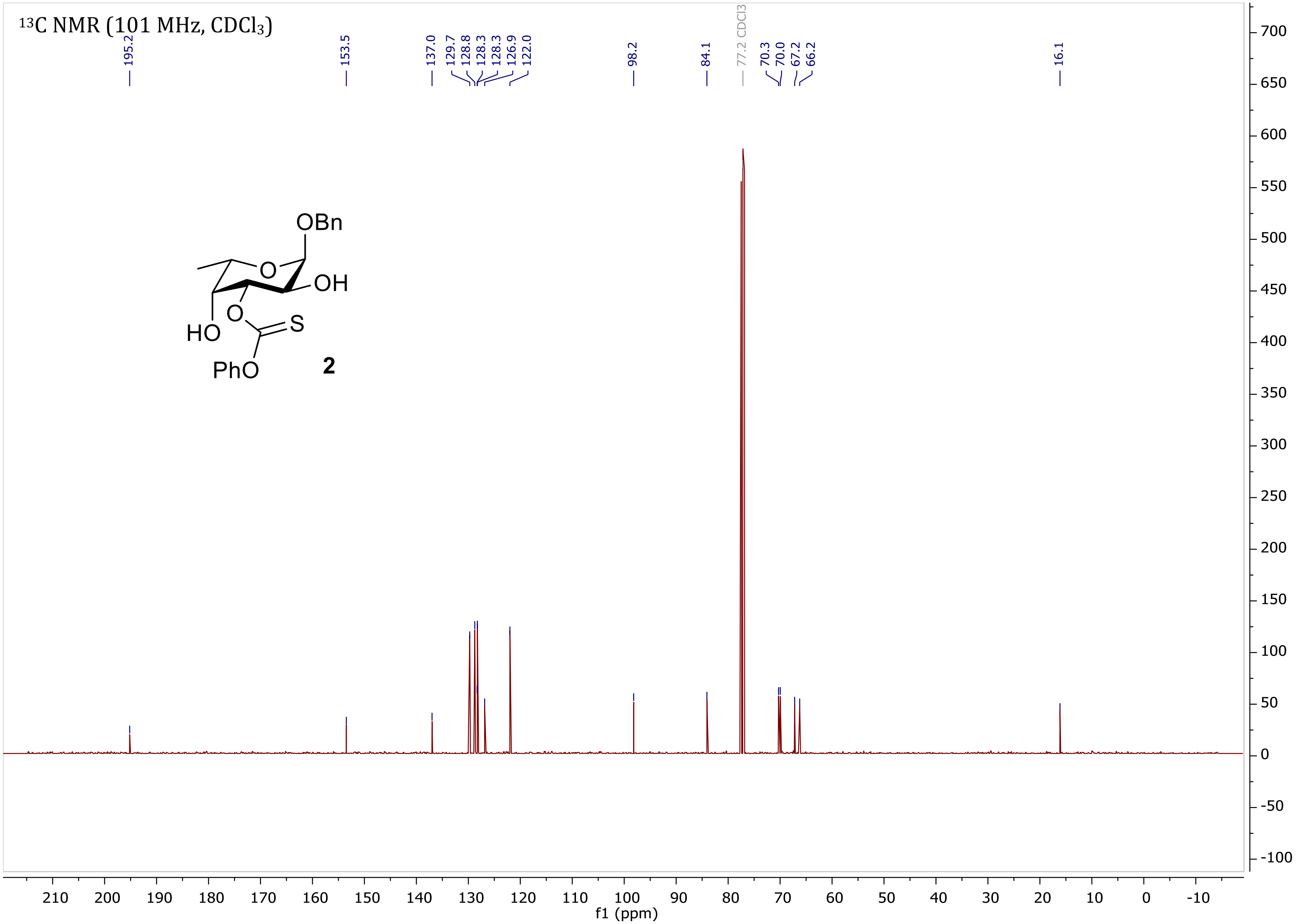

13C NMR (101 MHz, CDCl3)
195.2
153.5
137.0
129.7
128.8
128.3
128.3
126.9
122.0
98.2
84.1
77.2 CDCl3
70.3
70.0
67.2
66.2
16.1
OBn
O
OH
HO
O
S
PhO
2
f1 (ppm)

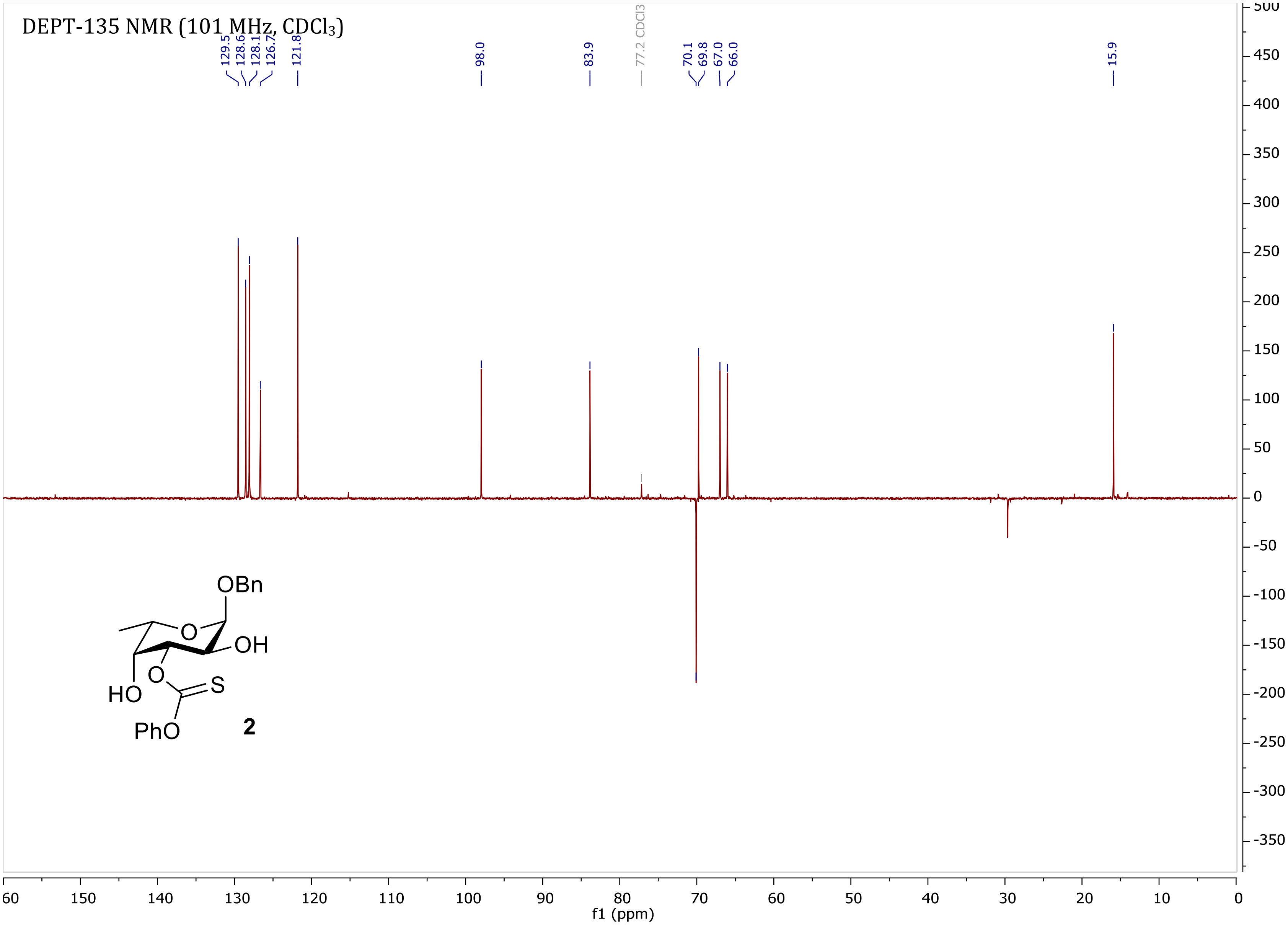
DEPT-135 NMR (101 MHz, CDCl3)
129.5
128.6
128.1
126.7
121.8
98.0
83.9
77.2 CDCl3
70.1
69.8
67.0
66.0
15.9
OBn
O
OH
HO
O
S
PhO
2
f1 (ppm)

NMR of Benzyl 3-deoxy-α-L-fucopyranoside (3)

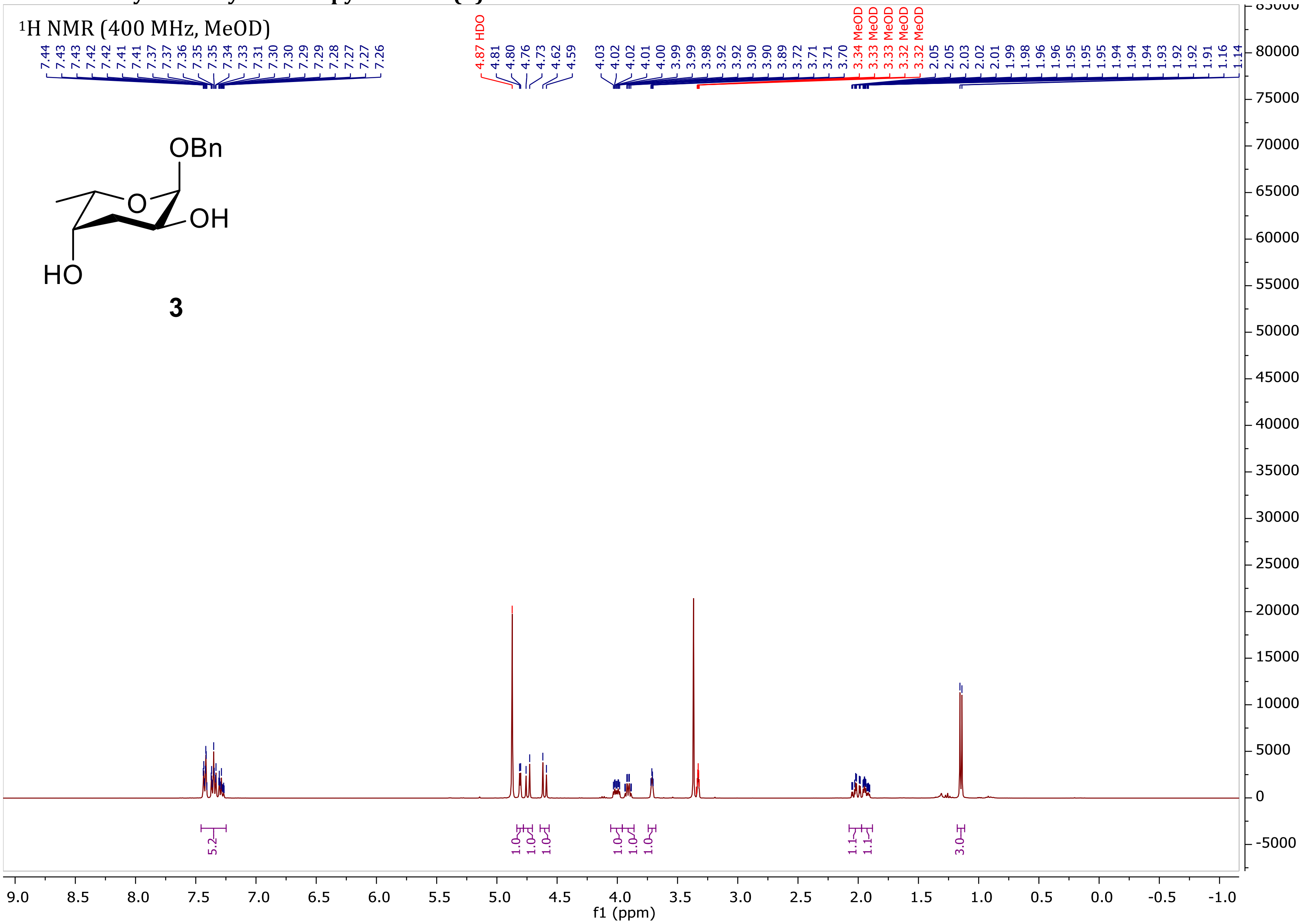

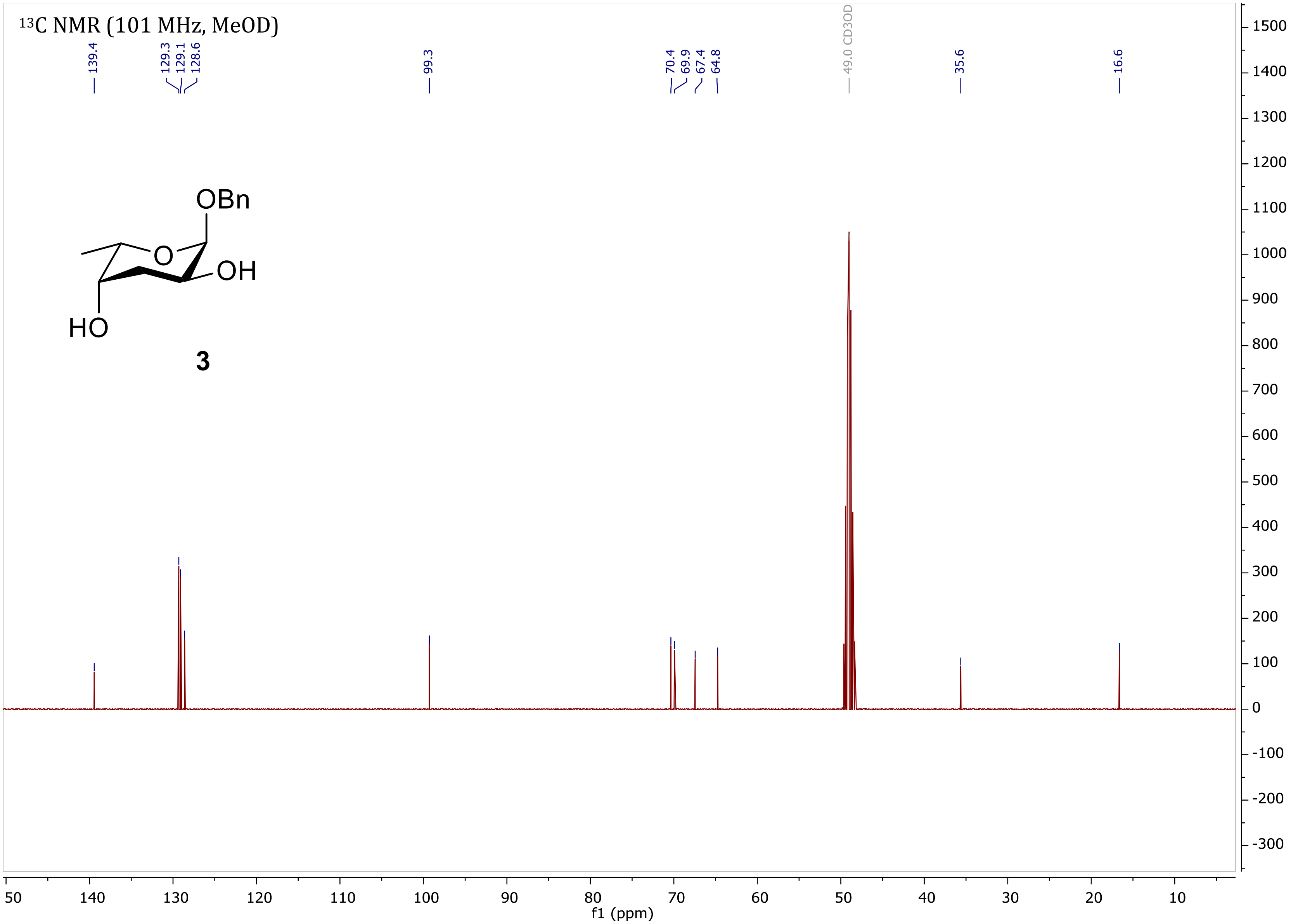

13C NMR (101 MHz, MeOD)
139.4
129.3
129.1
128.6
99.3
70.4
69.9
67.4
64.8
49.0 CD3OD
35.6
16.6
OBn
O
OH
HO
3
f1 (ppm)

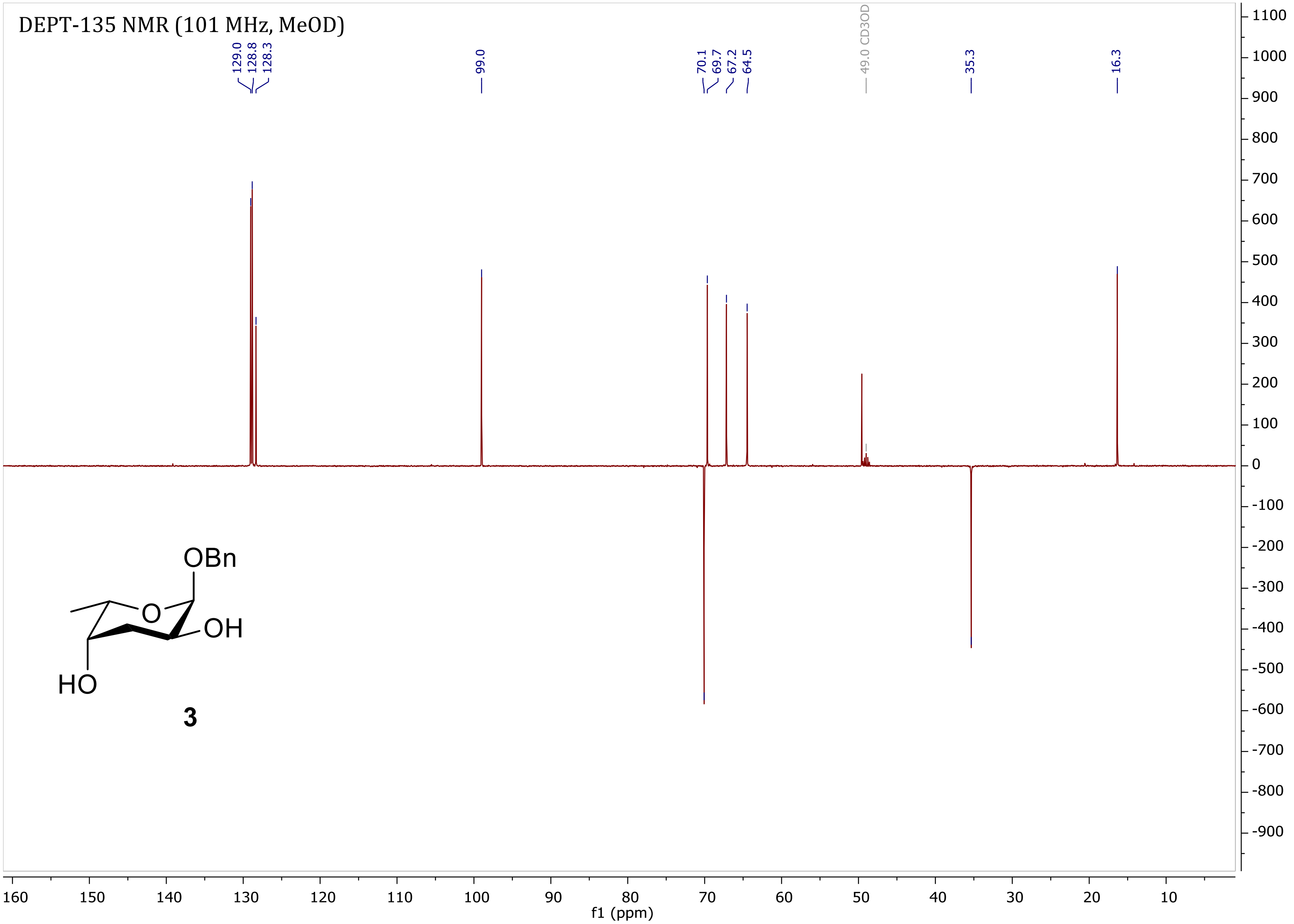
DEPT-135 NMR (101 MHz, MeOD)
129.0
128.8
128.3
99.0
70.1
69.7
67.2
64.5
49.0 CD3OD
35.3
16.3
f1 (ppm)
OBn
O
OH
HO
3

NMR of L-colitose (4)

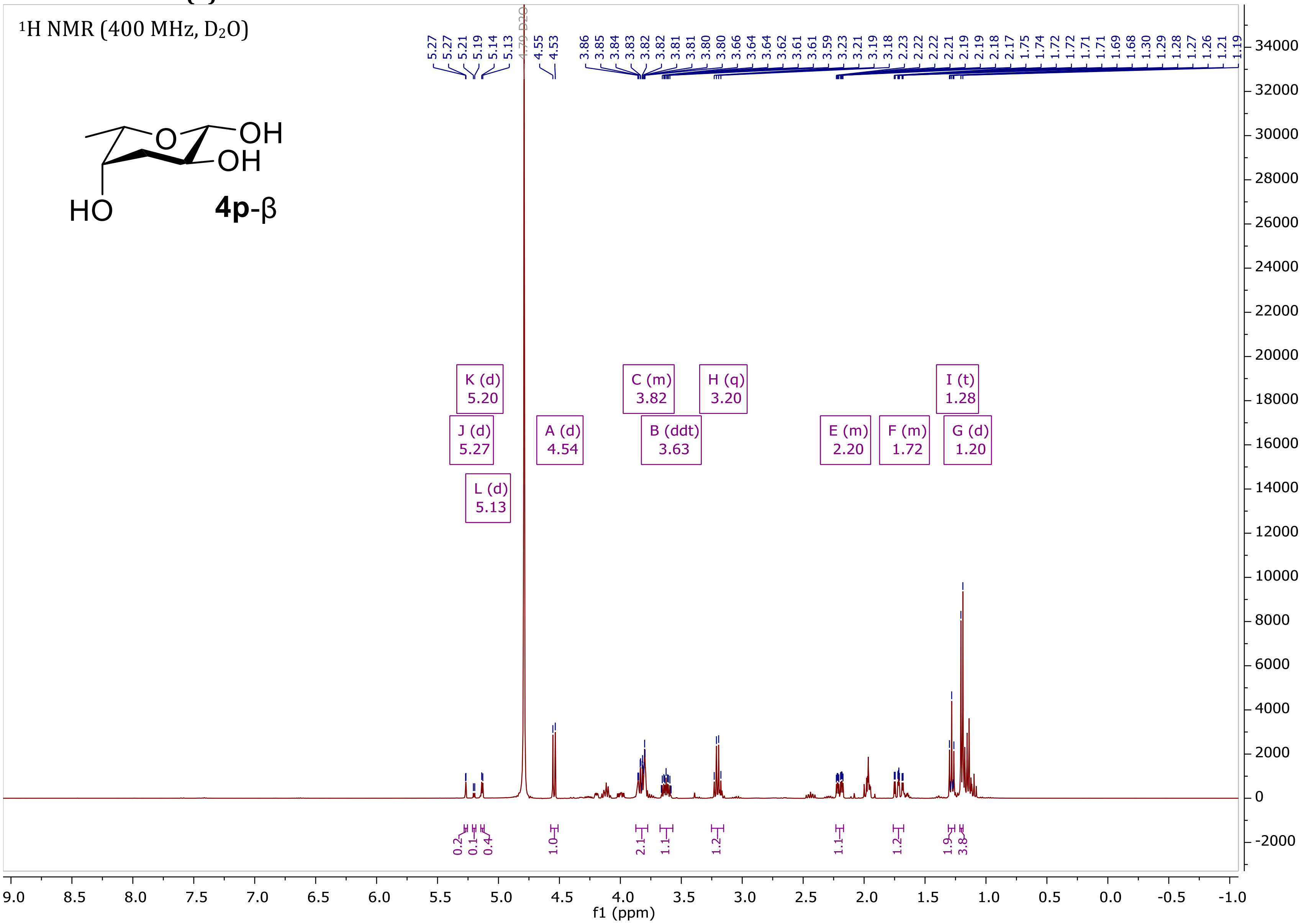

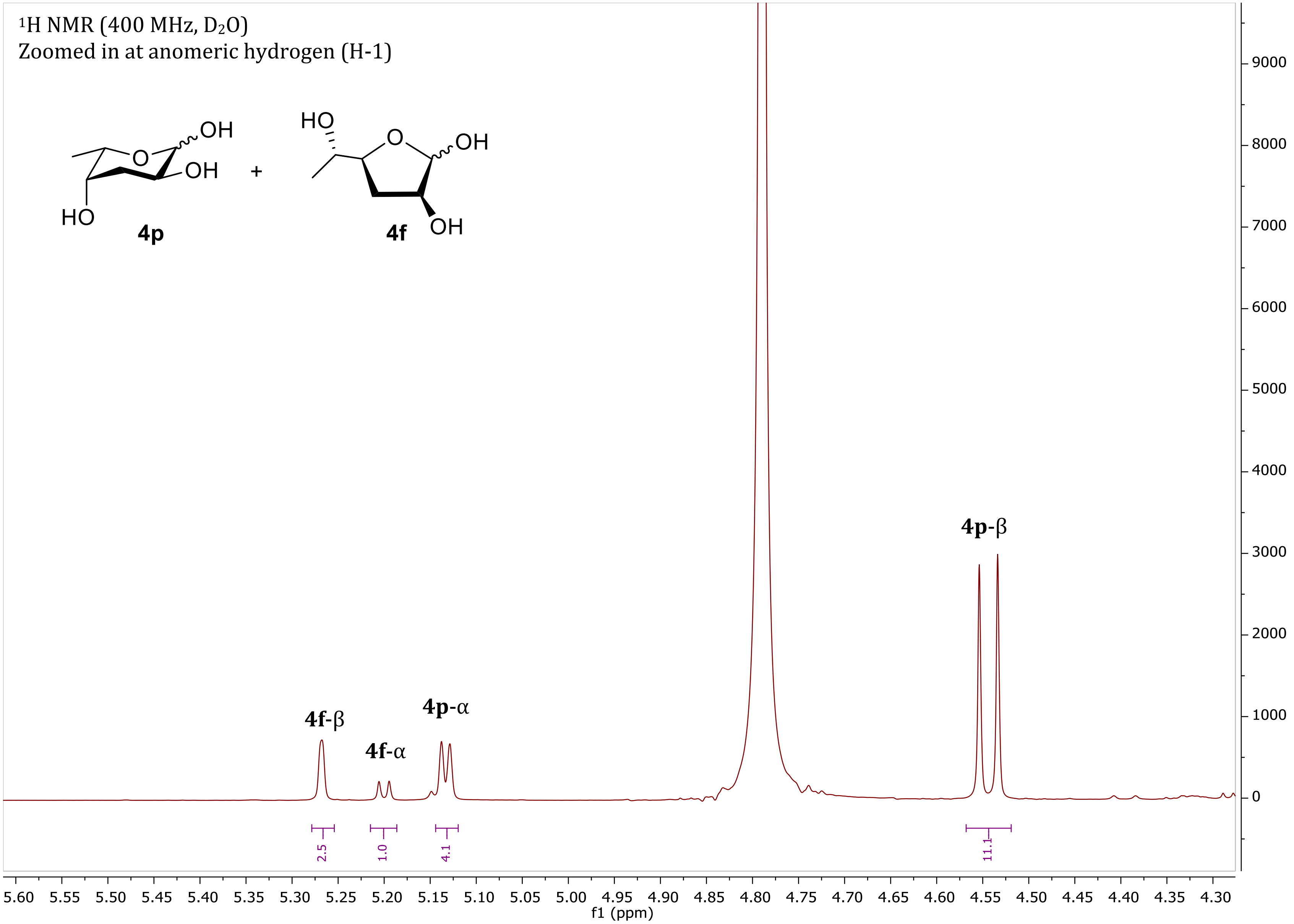

1H NMR (400 MHz, D2O)
Zoomed in at anomeric hydrogen (H-1)
OH
O
OH
HO
4p
+
HO
O
OH
4f
OH
4f-β
4f-α
4p-α
4p-β
2.5
1.0
4.1
11.1
f1 (ppm)

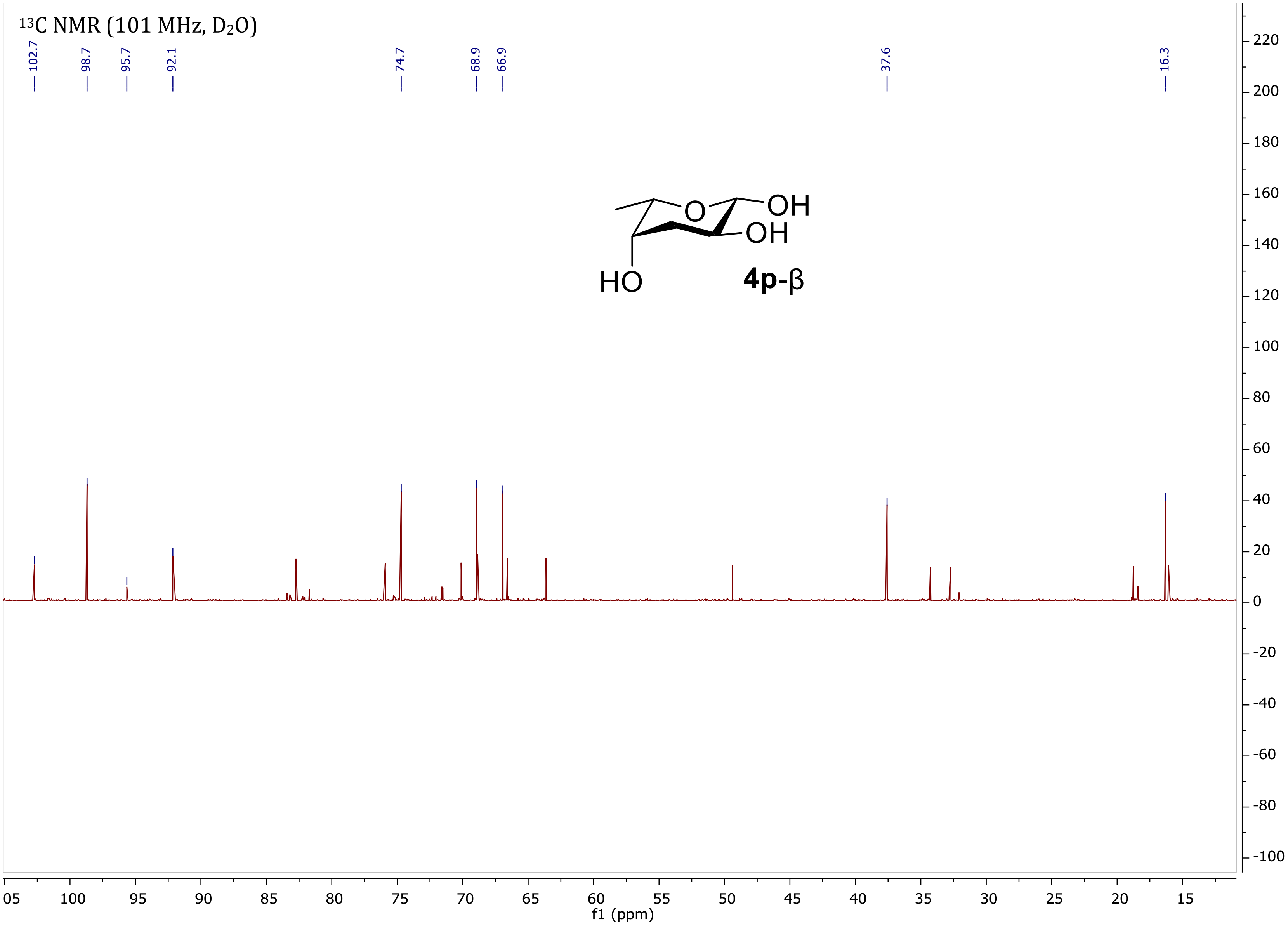

13C NMR (101 MHz, D2O)
102.7
98.7
95.7
92.1
74.7
68.9
66.9
37.6
16.3
OH
OH
O
HO
4p-β
f1 (ppm)

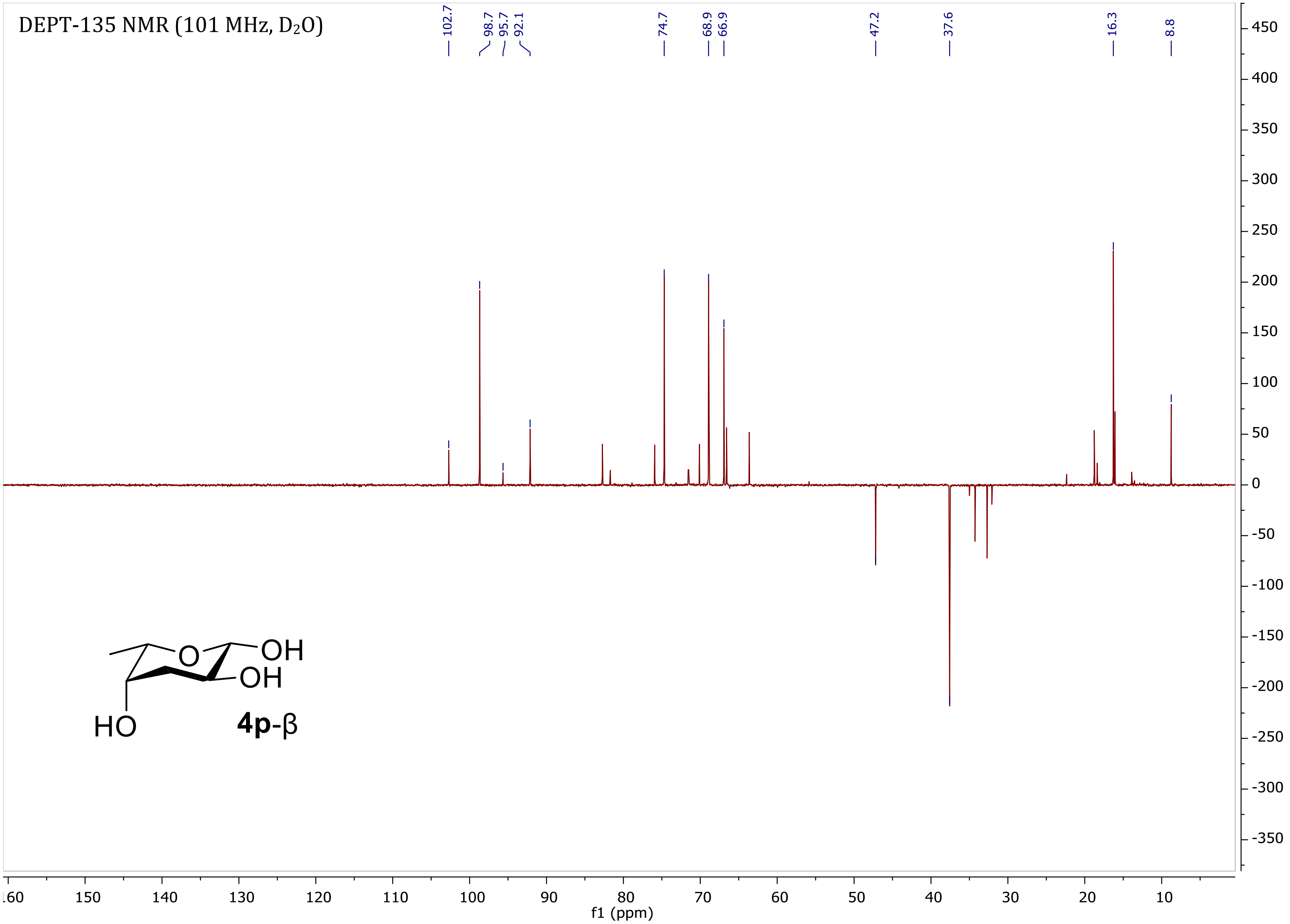

DEPT-135 NMR (101 MHz, D₂O)
102.7
98.7
95.7
92.1
74.7
68.9
66.9
47.2
37.6
16.3
8.8
OH
OH
O
HO
4p-β
f1 (ppm)

References:

1. Ireland, R. E.; Anderson, R. C.; Badoud, R.; Fitzsimmons, B. J.; McGarvey, G. J.; Thaisrivongs, S.; Wilcox, C. S., The total synthesis of ionophore antibiotics. A convergent synthesis of lasalocid A (X537A). *J Am Chem Soc* **1983,** *105* (7), 1988-2006.
2. Greene, W. T.; Wuts, G. M. P., The Role of Protective Groups in Organic Synthesis. In *Protective Groups in Organic Synthesis*, 3rd ed; John Wiley & Sons, 1999; pp 1-16.
3. Lloyd, D.; Bennett, C. S., Gram-Scale Synthesis of an Armed Colitose Thioglycoside. *J Org Chem* **2014,** *79* (20), 9826-9829.
4. Muramatsu, W.; Tanigawa, S.; Takemoto, Y.; Yoshimatsu, H.; Onomura, O., Organotin-Catalyzed Highly Regioselective Thiocarbonylation of Nonprotected Carbohydrates and Synthesis of Deoxy Carbohydrates in a Minimum Number of Steps. *Chem Eur J* **2012,** *18* (16), 4850-4853.
5. Lindhorst, T. K.; Thiem, J., The synthesis of 3-deoxy-L-fucose (3,6-dideoxy-L-xylo-hexose). *Liebigs Ann Chem* **1990,** (12), 1237-1241.
6. Zunk, M.; Kiefel, M. J., An efficient synthesis of selectively functionalized D-rhamnose derivatives. *Tetrahedron Lett* **2011,** *52* (12), 1296-1299.
7. Jumper, J.; Evans, R.; Pritzel, A.; Green, T.; Figurnov, M.; Ronneberger, O.; Tunyasuvunakool, K.; Bates, R.; Žídek, A.; Potapenko, A.; Bridgland, A.; Meyer, C.; Kohl, S. A. A.; Ballard, A. J.; Cowie, A.; Romera-Paredes, B.; Nikolov, S.; Jain, R.; Adler, J.; Back, T.; Petersen, S.; Reiman, D.; Clancy, E.; Zielinski, M.; Steinegger, M.; Pacholska, M.; Berghammer, T.; Bodenstein, S.; Silver, D.; Vinyals, O.; Senior, A. W.; Kavukcuoglu, K.; Kohli, P.; Hassabis, D., Highly accurate protein structure prediction with AlphaFold. *Nature* **2021,** *596* (7873), 583-589.
8. Varadi, M.; Bertoni, D.; Magana, P.; Paramval, U.; Pidruchna, I.; Radhakrishnan, M.; Tsenkov, M.; Nair, S.; Mirdita, M.; Yeo, J.; Kovalevskiy, O.; Tunyasuvunakool, K.; Laydon, A.; Žídek, A.; Tomlinson, H.; Hariharan, D.; Abrahamson, J.; Green, T.; Jumper, J.; Birney, E.; Steinegger, M.; Hassabis, D.; Velankar, S., AlphaFold Protein Structure Database in 2024: providing structure coverage for over 214 million protein sequences. *Nucleic Acids Res* **2024,** *52* (D1), D368-d375.
9. Schrodinger, LLC, The PyMOL Molecular Graphics System, Version 1.8. 2015.
10. Wu, Z.; Zhao, G.; Li, T.; Qu, J.; Guan, W.; Wang, J.; Ma, C.; Li, X.; Zhao, W.; Wang, P. G.; Li, L., Biochemical characterization of an α1,2-colitosyltransferase from *Escherichia coli* O55:H7. *Glycobiol* **2016,** *26* (5), 493-500.
11. Hirayama, K.; Akashi, S.; Furuya, M.; Fukuhara, K., Rapid confirmation and revision of the primary structure of bovine serum albumin by ESIMS and frit-FAB LC/MS. *Biochem Biophys Res Comm* **1990,** *173* (2), 639-646.
12. Coyne, M. J.; Reinap, B.; Lee, M. M.; Comstock, L. E., Human symbionts use a host-like pathway for surface fucosylation. *Science* **2005,** *307* (5716), 1778-81.
13. Hacker, S. M.; Welter, M.; Marx, A., Synthesis of γ-Phosphate-Labeled and Doubly Labeled Adenosine Triphosphate Analogs. *Curr Protoc Nucleic Acid Chem* **2015,** *60*, 13.14.1-13.14.25.
14. Bryksin, A. V.; Matsumura, I., Overlap extension PCR cloning: a simple and reliable way to create recombinant plasmids. *BioTechniques* **2010,** *48* (6), 463-465.
15. Yi, W.; Liu, X.; Li, Y.; Li, J.; Xia, C.; Zhou, G.; Zhang, W.; Zhao, W.; Chen, X.; Wang, P. G., Remodeling bacterial polysaccharides by metabolic pathway engineering. *Proc Natl Acad Sci U S A* **2009,** *106* (11), 4207-12.
